# Functional States in Tumor-Initiating Cell Differentiation in Human Colorectal Cancer

**DOI:** 10.3390/cancers13051097

**Published:** 2021-03-04

**Authors:** Martina K. Zowada, Stephan M. Tirier, Sebastian M. Dieter, Teresa G. Krieger, Ava Oberlack, Robert Lorenz Chua, Mario Huerta, Foo Wei Ten, Karin Laaber, Jeongbin Park, Katharina Jechow, Torsten Müller, Mathias Kalxdorf, Mark Kriegsmann, Katharina Kriegsmann, Friederike Herbst, Jeroen Krijgsveld, Martin Schneider, Roland Eils, Hanno Glimm, Christian Conrad, Claudia R. Ball

**Affiliations:** 1 Translational Functional Cancer Genomics, National Center for Tumor Diseases (NCT) Heidelberg and German Cancer Research Center (DKFZ) Heidelberg, 69120 Heidelberg, Germany; martina.zowada@nct-heidelberg.de (M.K.Z.); sebastian.dieter@nct-heidelberg.de (S.M.D.); ava.oberlack@med.uni-muenchen.de (A.O.); mario.huerta@nct-heidelberg.de (M.H.); karin.laaber@nct-heidelberg.de (K.L.); friederike.herbst@nct-heidelberg.de (F.H.); 2 Department of Translational Medical Oncology, NCT Dresden and DKFZ, 01307 Dresden, Germany; 3 Faculty of Biosciences, Heidelberg University, 69120 Heidelberg, Germany; 4 Division of Theoretical Bioinformatics, DKFZ Heidelberg, 69120 Heidelberg, Germany; s.tirier@dkfz-heidelberg.de (S.M.T.); teresa.krieger@charite.de (T.G.K.); robert-lorenz.chua@charite.de (R.L.C.); foo-wei.ten@charite.de (F.W.T.); j.park@dkfz-heidelberg.de (J.P.); katharina.jechow@bihealth.de (K.J.); roland.eils@charite.de (R.E.); 5 Center for Quantitative Analysis of Molecular and Cellular Biosystems (BioQuant), Heidelberg University, 69120 Heidelberg, Germany; 6 Division of Chromatin Networks, DKFZ Heidelberg, 69120 Heidelberg, Germany; 7 German Cancer Consortium (DKTK), 69120 Heidelberg, Germany; 8 Digital Health Center, Berlin Institute of Health (BIH) and Charité-Universitätsmedizin Berlin, 10117 Berlin, Germany; 9 Division of Proteomics of Stem Cells and Cancer, DKFZ Heidelberg, 69120 Heidelberg, Germany; torsten.mueller@dkfz-heidelberg.de (T.M.); mathiaskalxdorf@gmail.com (M.K.); j.krijgsveld@dkfz-heidelberg.de (J.K.); 10 Medical Faculty, Heidelberg University, 69120 Heidelberg, Germany; 11 Institute of Pathology, Heidelberg University Hospital, 69120 Heidelberg, Germany; Mark.Kriegsmann@med.uni-heidelberg.de; 12 Department of Hematology, Oncology and Rheumatology, Heidelberg University Hospital, 69120 Heidelberg, Germany; Katharina.Kriegsmann@med.uni-heidelberg.de; 13 Department of General, Visceral and Transplantation Surgery, Heidelberg University Hospital, 69120 Heidelberg, Germany; Martin.Schneider@med.uni-heidelberg.de; 14 Department for Bioinformatics and Functional Genomics, Institute for Pharmacy and Molecular Biotechnology (IPMB), Heidelberg University, 69120 Heidelberg, Germany; 15 Center for Personalized Oncology, University Hospital Carl Gustav Carus Dresden at Technische Universität (TU) Dresden, 01307 Dresden, Germany; 16 DKTK, 01307 Dresden, Germany

**Keywords:** colorectal cancer, tumor-initiating cells, tumor heterogeneity, patient-derived cancer models, single-cell RNA-sequencing, tumor metabolism, transcriptional programs, tumor cell differentiation

## Abstract

**Simple Summary:**

Different types of cells with tumor-initiating cell (TIC) activity contribute to colorectal cancer (CRC) progression and resistance to anti-cancer treatment. In this study, we aimed to understand whether different cell types exist within a patient-derived tumor culture, distinguishable by different patterns of their gene expression. By mRNA sequencing of patient-derived CRC cultures at the single-cell level, we defined expression programs that closely resemble differentiated cell populations of the normal intestine. Here, cell type-associated subpopulations showed differences in functional properties such as cell growth and energy metabolism. Subsequent functional analyses in vitro and in vivo demonstrated that metabolic states are linked to TIC activity in primary CRC cultures. We also show that TIC activity is dependent on oxidative phosphorylation, which may therefore represent a target for novel therapies.

**Abstract:**

Intra-tumor heterogeneity of tumor-initiating cell (TIC) activity drives colorectal cancer (CRC) progression and therapy resistance. Here, we used single-cell RNA-sequencing of patient-derived CRC models to decipher distinct cell subpopulations based on their transcriptional profiles. Cell type-specific expression modules of stem-like, transit amplifying-like, and differentiated CRC cells resemble differentiation states of normal intestinal epithelial cells. Strikingly, identified subpopulations differ in proliferative activity and metabolic state. In summary, we here show at single-cell resolution that transcriptional heterogeneity identifies functional states during TIC differentiation. Furthermore, identified expression signatures are linked to patient prognosis. Targeting transcriptional states associated to cancer cell differentiation might unravel novel vulnerabilities in human CRC.

## 1. Introduction

In many tumor entities, tumor formation and progression are driven by a cellular subfraction with tumor-initiating cell (TIC) activity [1,2,3]. In colorectal cancer (CRC), the TIC compartment is organized as a functional cellular hierarchy with extensively self-renewing long-term TICs driving serial tumor propagation in vivo. Long-term TICs generate highly proliferative, short-lived tumor transient-amplifying cells with limited or no self-renewal capacity giving rise to the bulk of post-mitotic tumor cells [4]. Remarkably, this functional heterogeneity within individual CRCs is not primarily driven by genetic events, suggesting that epigenetic or extrinsic factors contribute to functional cellular heterogeneity [5].

Lineage-tracing experiments demonstrate that in CRC the population of highly self-renewing TICs expresses *LGR5* and generates progeny differentiating towards mucosecreting- and absorptive-like phenotypes [6]. Thus, CRCs harbor a subfraction of stem-like TICs and maintain a hierarchical organization reminiscent of the normal intestinal epithelium [7]. Moreover, a gene signature specific for intestinal stem cells has been suggested to predict disease relapse [8], indicating a potential clinical relevance of stem-like TICs for CRC patients. However, prospective validation in an independent cohort is still not available.

Recent evidence suggests that the epigenome of an individual CRC is already formed by the cell-of-origin. Methylation analyses demonstrate maintenance of the cell-of-origin differentiation state during tumor progression, and identified three CRC subclasses of intestinal crypt differentiation of the cell-of-origin. Importantly, patients with a stem-like methylation signature showed significantly reduced overall survival [9].

While the hierarchical organization of normal and malignant stem cell systems has previously been thought to be fixed and unidirectional, evidence for plasticity in these systems is accumulating [10,11,12]. Lineage-tracing experiments in CRC highlight that more differentiated cells can repopulate a free stem-like niche and acquire TIC activity upon ablation of the active stem-like population [6,13,14]. Similarly, pronounced plasticity drives pancreatic cancer by clonal succession of transient TIC activity [15].

Current understanding of TIC heterogeneity in CRC is mainly derived from serial syngeneic or xenogeneic transplantation models, where TICs have been retrospectively identified by interpreting the kinetics of genetically marked or pre-enriched bulk cells [4,8,16,17,18]. While this allowed deep insights into functional heterogeneity within tumors, such retrospective experimental strategies from bulk samples hamper direct assignment of transcriptional states in individual cells.

To characterize molecular underpinnings of functional CRC intra-tumor heterogeneity at the single-cell level, we here asked whether distinct functional programs within individual cells from patient-derived CRC models can be assigned to specific cellular subpopulations.

## 2. Results

### 2.1. Transcriptional Heterogeneity of Patient-Derived CRC Spheroid Cultures

To assess whether heterogeneous transcriptional programs can be detected in CRC tumor spheroids at the single-cell level, we performed single-cell RNA-sequencing (scRNA-seq) using a nanowell platform [19]. As patient-derived spheroid cultures contain purely tumor cells, thereby allowing to study tumor cell heterogeneity in high resolution, and recapitulate the histology of the original tumor after xenotransplantation into immunodeficient NOD.Cg-*Prkdc^scid^Il2rg^tm1Wjl^*/SzJ (NSG) mice [4], we sequenced 12 three-dimensional tumor spheroid cultures (P1–P12) derived from primary tumors (*n* = 6 patients) or metastases (*n* = 6 patients) of 12 different CRC patients. These patient tumors and derived spheroids cover known subtypes (microsatellite stable or microsatellite instable tumors) and driver mutations (loss of *APC* and/or *TP53*, activating mutations in *KRAS*; Table 1). On average, 389 cells (range: 141–736) were sequenced per patient, resulting in 4663 single-cell profiles with an average of more than 4000 detected genes per cell (Table 2).

**Figure 1:**
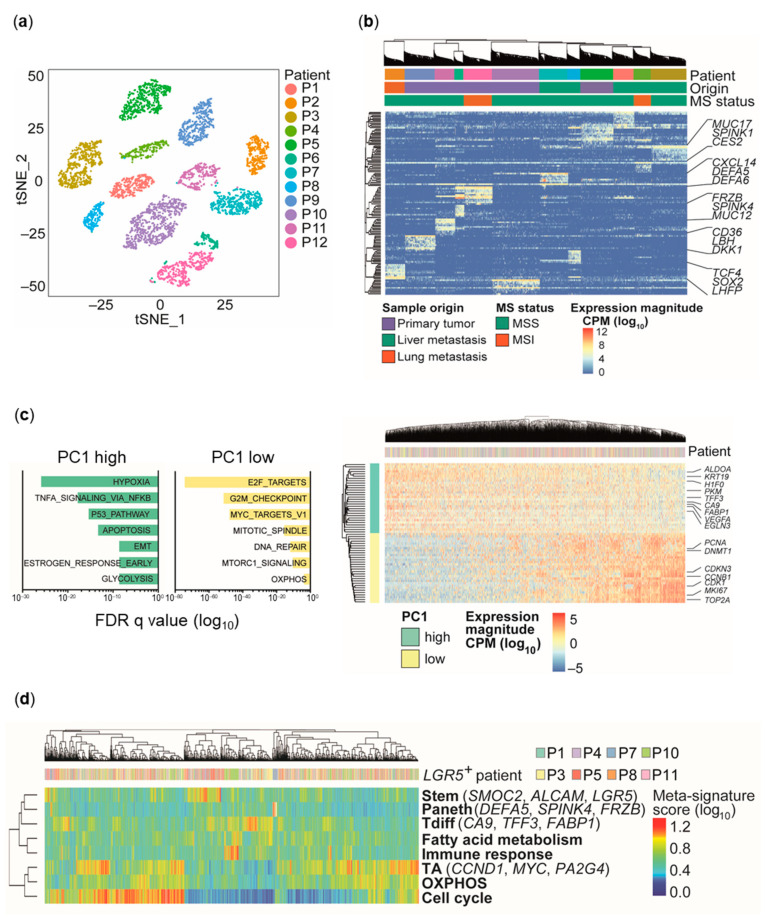
Identification of transcriptional subpopulations by single-cell RNA-sequencing (scRNA-seq). (**a**) Two-dimensional t-distributed stochastic neighbor embedding (tSNE) visualization of scRNA-seq expression profiles. (**b**) Hierarchical clustering and heatmap visualization of single-cell gene expression using the top 10 differentially expressed genes per patient (*n* = 12). CPM, counts-per-million; MS, microsatellite; MSI, microsatellite instable; MSS, microsatellite stable. (**c**) Principal component analysis of scRNA-seq data corrected for inter-patient variability. Left: Gene set enrichment analysis (GSEA) for the first principal component (PC1; hallmark gene sets). Gene sets are ranked by false discovery rate (FDR) q values. Right: Heatmap showing gene expression magnitude of the top 30 genes with highest and lowest PC scores for PC1. OXPHOS, oxidative phosphorylation. (**d**) Heatmap reflecting hierarchical clustering of core meta-signature scores of eight *LGR5*^+^ CRC spheroid cultures determined by non-negative matrix factorization. Brackets indicate marker genes for specific signatures. TA, transit-amplifying; Tdiff, terminally differentiated.

Unsupervised clustering of single-cell profiles [20] revealed grouping of cells according to the patient-of-origin (Figure 1a). Hierarchical clustering based on the top 10 differentially expressed genes per patient showed that cells primarily cluster, with one exception, by the tumor site they originate from, but not by microsatellite status (Figure 1a,b).

Within individual patient-derived spheroids, top differentially expressed genes (Wilcoxon rank sum test: adjusted *p*-value < 0.05; log fold-change > 0.25) between patients contained WNT signaling components and downstream targets (e.g., *FRZB*, *DKK1*, *TCF4*, *SOX2*) and normal tissue-associated differentiation markers (e.g., *MUC12*, *MUC17*, *SPINK1*, *SPINK4*, *DEFA5*, *DEFA6*; Figure 1b). Thus, beyond patient tumor-specific alterations, differentiation state-associated expression programs can be attributed to transcriptional profiles derived from single CRC cells.

### 2.2. Distinct Cell Types and Cell States in Individual CRC Spheroids

To identify heterogeneous gene expression programs shared across patients in single cells from individual tumor spheroid cultures, we corrected for inter-patient variability by calculating relative expression levels for each patient individually [21,22]. Principal component analysis (PCA) of the combined dataset revealed an anti-correlated transcriptional pattern independent of patient origin with genes either involved in cell growth, proliferation, and oxidative phosphorylation (OXPHOS), or hypoxia and glycolysis. Notably, the hypoxia/glycolysis signature contains intestinal differentiation markers (e.g., *TFF3*, *FABP1*, *KRT19*; Figure 1c), indicating an association of distinct metabolic states with tumor cell differentiation and proliferation, as recently described for the normal intestinal crypt [23].

As the activation of continuous gene expression programs may not be captured by discrete clustering, we adapted a previously described computational approach based on non-negative matrix factorization (NNMF) [24,25] to more precisely identify transcriptional programs heterogeneously expressed across patients (Figure 1d, Figure S1a–e). In order to focus on tumors that display preserved hierarchical organization, we focused on the eight cultures with detectable *LGR5* transcript levels (*LGR5* score = total *LGR5* transcript counts/cell number > 1; Table 2), as *LGR5* represents an established marker for intestinal stem cells and CRC TICs, whereas the phenotype and the role of potential *LGR5*-negative stem cells and TICs are much less defined [8,14,26,27]. Thus, four cultures with very low or non-detectable *LGR5* transcript abundance were excluded for this analysis. We identified 13 heterogeneous gene expression programs that could be classified into two partially overlapping categories: one (A) linked to ‘cell types’ or lineages analogous to the normal intestinal epithelium, and the other (B) associated with ‘cell states’ (Figure S1f).

Category A identified distinct cells harboring marker expression similarities to normal intestinal stem cells (e.g., *LGR5*, *AXIN2*), Paneth cells (e.g., *DEFA5*, *DEFA6*), or transit-amplifying (TA) cells (e.g., *PA2G4*, *CCND1*) in the healthy human intestine, suggesting that distinct cell types can be identified based on individual gene expression programs. As the analyzed cells derive from the colon and only resemble the cell types of the normal intestine, we refer to these cell type-associated subpopulations as stem-like, Paneth-like, TA-like, and terminally differentiated (Tdiff)-like. Category B comprised expression programs enriched for genes involved in cell cycle regulation (e.g., *CDK1*, *MKI67*), immune/stress response (e.g., *CEACAM6*, *CXCL2*), or metabolic functions (e.g., OXPHOS (e.g., *PRDX3*, *ATP5O*), fatty acid metabolism (e.g., *CES2*, *RETSAT*), and hypoxia/glycolysis (e.g., *HILPDA*, *VEGFA*)). Similar to PCA results (Figure 1c), one expression program (Tdiff) was enriched for both, genes associated with hypoxia/glycolysis and differentiation markers (e.g., *TFF3*, *KRT20*; Figure S1f).

Next, each individual cell was scored for inferred expression programs using the averaged expression of the top genes per factor identified by NNMF. To reduce redundancy, signatures showing similar enrichment and clustering patterns were combined, resulting in eight meta-signatures (Figure S1a–f, Table S1). Clustering of meta-signature scores allowed identification of discrete and overlapping transcriptional programs (Figure 1d). Similar to PCA, cell cycle, OXPHOS, and TA signatures showed a pronounced overlap, indicating a highly proliferative cell fraction—potentially corresponding to the TA-like compartment—driven by MYC and characterized by high OXPHOS. In contrast, stem-like, Paneth-like, and Tdiff-like cells did not show significant overlap with the cell cycle signature (Figure 1d), suggesting reduced or absent proliferative activity. This indicates that scRNA-seq and matrix factorization analysis are capable of distinguishing functionally distinct cell populations based on transcriptional profiles.

To analyze the cell type composition in all eight *LGR5*^+^ cultures individually, we used the NNMF-inferred signature scores (stem, TA, Paneth, Tdiff) to assign cells to one of the four cell types which allowed us to assess the extent of active cell type-specific transcriptional programs. Despite different cell type compositions, we observed presence of stem-like, TA-like, and Tdiff-like cells in all, and rare, but detectable Paneth-like cells in six out of the eight *LGR5*^+^ cultures (Figure S1g). This indicates that individual CRC tumors display similar cellular diversity resembling normal intestinal cell types even with different clinico-pathological features (Table 1).

**Figure 2:**
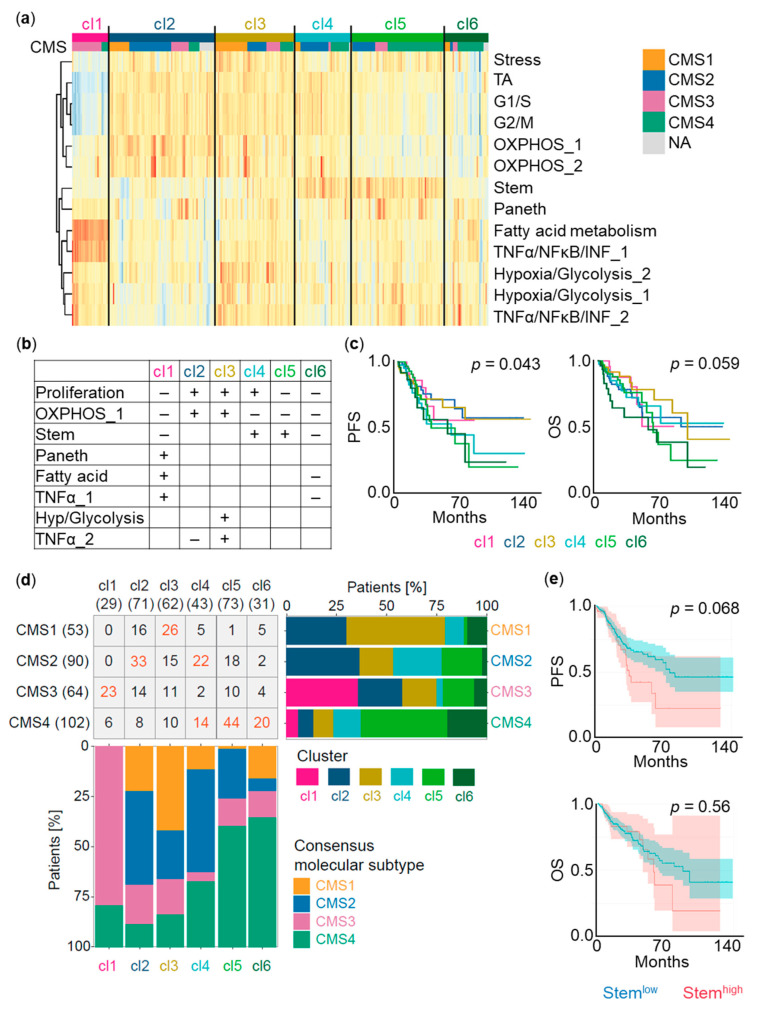
Analysis of single-cell RNA-sequencing (scRNA-seq) signature expression in The Cancer Genome Atlas (TCGA) colon adenocarcinoma (COAD) cohort. (**a**) Heatmap reflecting signature expression in the identified clusters of patients (cl1–cl6). Rows correspond to scRNA-seq signatures (*n* = 13). Columns correspond to TCGA COAD samples (*n* = 328). Clusters are further classified according to consensus molecular subtypes (CMS1–CMS4). NA, not assigned; OXPHOS, oxidative phosphorylation; TA, transit-amplifying. (**b**) Cell type and cell state signature expression levels defining cl1–cl6. Hyp, hypoxia. (**c**) Kaplan–Meier survival curves displaying progression-free survival (PFS) and overall survival (OS) for cl1–cl6. *p*-Values of the comparison cl6 + cl5 + cl4 versus cl3 + cl2 + cl1 for PFS and cl6 + cl5 versus cl4 + cl3 + cl2 + cl1 for OS. (**d**) Representation of CMS1–CMS4 within cl1–cl6 and of cl1–cl6 within CMS1–CMS4. Numbers indicate amount of patients classified under individual categories. Numbers marked in red highlight dominant combinations. Patients not assigned to a CMS (*n* = 19 patients) were excluded. (**e**) PFS and OS of TCGA COAD patients with high versus low expression of the stem signature. Shaded areas indicate 95% confidence intervals.

We next assessed whether the signatures identified in our patient-derived in vitro models can also be identified in patient tumors. We therefore applied our signatures (Table S1) on publicly available expression data of colon adenocarcinoma (COAD) patients (The Cancer Genome Atlas (TCGA) cohort; *n* = 328 patients) [28]. Correlations among cell type and cell state signatures in the spheroid scRNA-seq data (Figure 1d) were detectable in patient whole transcriptome data. Clustering of the TCGA cohort based on signature expression resulted in six clusters of patients (cl1–cl6) with different combinations of low or high expression of individual signatures. Significantly different progression-free survival (*p* = 0.043) and numerically decreased overall survival (*p* = 0.059) were observed between groups of clusters, indicating a relevance of signature expression for patient prognosis (Figure 2a–c, Table S2).

We further compared the association of cl1–cl6 with consensus molecular subtypes (CMS1–CMS4) [29,30]. CMS1 tumors were mostly represented in cl3 (49%), CMS2 tumors displayed mostly cl2 (37%) and cl4 (24%), CMS3 tumors were predominantly found in cl1 (36%). CMS4 tumors were spread across cl4 (14%), cl5 (43%), and cl6 (20%). CMS4 has been shown to have poor progression-free survival [30]. Accordingly, cl4, cl5, and cl6 (33%, 60%, and 65% CMS4 contribution, respectively) showed the worst progression-free survival. cl6 comprised the majority of patients with the shortest overall survival of CMS4, whereas cl4 displayed worse progression-free survival than cl1, cl2, and cl3 but similar overall survival (Figure 2c,d).

In line with previously published data reporting an intestinal stem cell-specific gene signature linked to LGR5 and EPHB2 expression related to CRC relapse [8], high expression of our stem signature defined by 200 genes (Table S1) in the TCGA cohort displayed decreased progression-free survival (*p* = 0.068) compared to patients with low expression (Figure 2e).

Taken together, our six clusters exhibit a better prognostic value for progression-free survival (*p* = 0.043) than previously reported subtypes linked to cancer-associated fibroblasts [31] (*p* = 0.15), CMSs [29,30] (*p* = 0.18), or our stem signature alone (*p* = 0.068). Indeed, when our clusters were added to multivariable clinico-molecular survival models, we still observed a significant discriminative contribution by our cluster combinations in predicting recurrence, but no significant contribution was appreciated when adding CMSs or cancer-associated fibroblasts to our model. On the other hand, incorporating stroma cells like cancer-associated fibroblasts can substantially improve the overall survival prediction (Table S2). These results underscore the relevance of combinations of cell type and cell state signature expression for COAD outcome, and demonstrate a prognostic value of cell type and cell state signatures inferred from spheroid single-cell transcriptomes.

### 2.3. Cell Cycle and Proliferative Activity of Human CRC Cells

scRNA-seq suggests the existence of cell types with different proliferative activity within individual spheroid cultures and stem-like, TA-like, and Tdiff-like subpopulations. We therefore asked whether subfractions of cells with different cell cycle and proliferative activity exist within CRC tumors in vivo and whether they are functionally relevant.

To assess proliferative heterogeneity of tumor cells, we utilized a genetic label-retaining strategy based on expression of tetracycline-regulated (Tet-off) histone 2B (H2B) green fluorescent protein (GFP) [32] (Figure S2a). Upon doxycycline addition, nuclear H2B-GFP expression is suppressed and subsequently diluted with each cell division, allowing identification of subpopulations according to proliferative history.

To evaluate whether proliferatively inactive cells within established tumors possess TIC capacity, we transduced tumor spheroid cultures derived from seven different patients with an H2B-GFP-encoding lentiviral vector prior to xenotransplantation into NSG mice (*n* = 14; 1–4 mice per culture). After successful tumor formation, H2B-GFP expression was suppressed by doxycycline administration for two weeks. Analysis of H2B-GFP expression in established tumors by flow cytometry revealed presence of fast (GFP^–^), slow (GFP^low^), and rare dividing (GFP^high^) cell fractions, demonstrating proliferative heterogeneity of CRC cells in vivo. To assess whether heterogeneously proliferating cell fractions are associated with TIC activity, cells from 12 out of 14 primary xenografts were sorted into fast, slow, and rare dividing subfractions and serially transplanted into secondary mice (*n* = 33). Importantly, all subfractions contained cells with TIC activity irrespective of transplanted cells’ proliferative history prior to re-transplantation (fast: 5/12; slow: 4/9; rare: 5/12 mice with tumors), showing that TIC activity is not strictly linked to proliferative active cell fractions but also present in proliferatively inactive populations within tumors (Figure S2a–c). In summary, these data show that proliferatively inactive TICs exist within established tumors in vivo. We therefore conclude that within individual tumors, TIC activity can be present in cells with heterogeneous proliferative activity and is therefore not restricted to a specific proliferative state of individual cells.

### 2.4. Divergent Cell Type-Associated Energy Metabolic Preferences

Prominent heterogeneously expressed transcriptional programs in individual spheroid cells were related to energy metabolism. Whereas a glycolysis/hypoxia signature could be assigned to Tdiff-like cells (Figure S1f), OXPHOS strongly overlapped with MYC-target and cell cycle signatures, both identifying cells belonging to the putative TA-like cell compartment (Figure 1c,d). Thus, we hypothesized that metabolic preferences distinguish functionally distinct cell subpopulations and focused on these for further validation.

Consistently, we observed clearly overlapping TA-like, OXPHOS, and cell cycle signatures (Figure 1d), but no obvious association between stem-like and OXPHOS or cell cycle signatures. Of note, in the normal intestinal epithelium, intestinal stem cells actively cycle and constantly produce progeny, but their relative abundance compared to non-cycling Tdiff cells is very low [33]. Thus, we reasoned that differential metabolic trends in stem-like and Paneth-like cells could be masked by much higher or lower expression of individual metabolic signatures in highly cycling cells or the rare dividing Tdiff-like subpopulation. To overcome this, we performed pairwise comparisons of cell state signatures across CRC subpopulations that resemble normal intestinal cell types as identified by differential NNMF signature expression.

**Figure 3:**
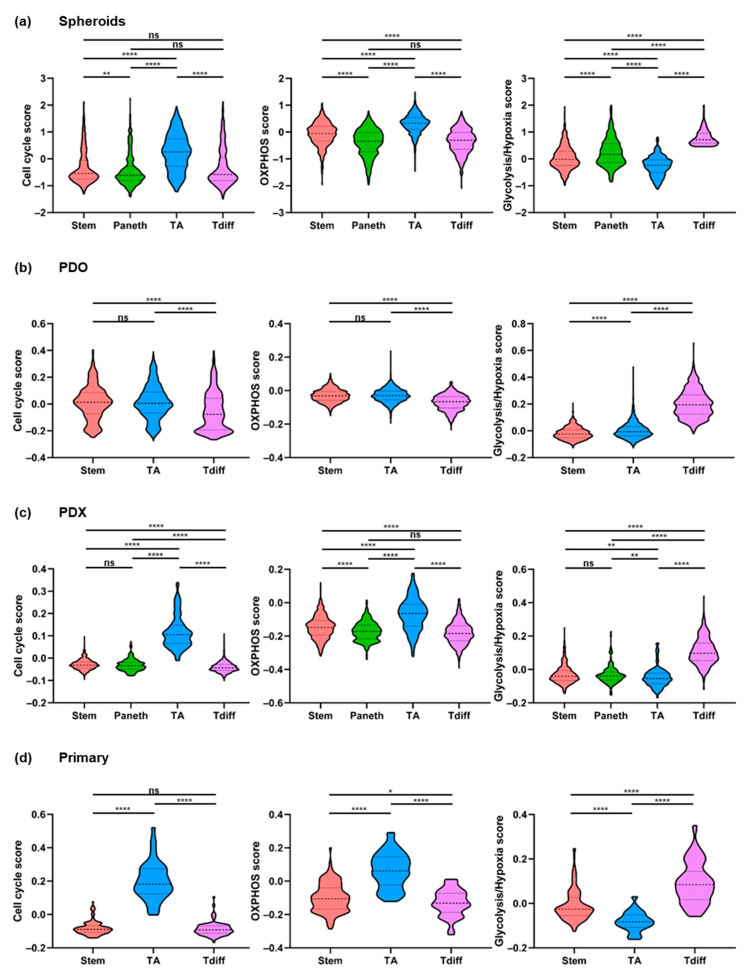
Cell state scores for cell type-specific cell subsets inferred by non-negative matrix factorization. (**a**–**d**) Cell cycle, oxidative phosphorylation (OXPHOS), and glycolysis/hypoxia scores (signatures G2/M, OXPHOS_2, and Hypoxia/Glycolysis_2, respectively) for cells of (**a**) spheroids (*n* = 8 *LGR5*^+^ cultures), (**b**) patient-derived organoid (PDO; O1), (**c**) patient-derived xenograft (PDX; X1), and (**d**) merged tumor epithelial cells (T1–T3) classified under active cell type-specific meta-signatures: stem-like (spheroids: *n* = 467; PDO: *n* = 944; PDX: *n* = 667; primary: *n* = 124), Paneth-like (spheroids: *n* = 357; PDX: *n* = 189), transit-amplifying (TA)-like (spheroids: *n* = 554; PDO: *n* = 3,967; PDX: *n* = 100; primary: *n* = 49), terminally differentiated (Tdiff)-like cells (spheroids: *n* = 486; PDO: *n* = 639; PDX: *n* = 424; primary: *n* = 80). *p*-Values were calculated based on the Mann–Whitney Test (two-tailed). * *p* < 0.05; ** *p* < 0.01; **** *p* < 0.0001; ns, not significant. Dashed lines indicate medians. Upper and lower dotted lines indicate 75% and 25% percentiles, respectively.

Cell cycle scores were strongly increased in TA-like cells compared to stem-like, Paneth-like, and Tdiff-like cells (*p* < 0.000001; respectively). The greatest differences in metabolic states existed between Tdiff-like and TA-like subpopulations, demonstrating that the majority of TA-like cells had high OXPHOS scores, whereas Tdiff-like cells showed high scores for hypoxia and glycolysis, but low scores for OXPHOS. Albeit less pronounced, similar and highly significant differences were detectable for stem-like and Paneth-like cells. In comparison to Paneth-like cells, stem-like cells showed increased OXPHOS scores and decreased glycolysis/hypoxia scores (*p* < 0.000001, respectively; Figure 3a).

In addition to the overall high OXPHOS scores, the stem-like signature was associated with enhanced expression of *OXR1* and *PON2*. Being essential for protection against oxidative stress, these genes may counteract higher reactive oxygen species (ROS) levels resulting from enhanced OXPHOS rates [34,35]. Another gene included in the stem-like signature is *MAP2K6*—an essential p38 signaling component [36] known to be associated with high OXPHOS levels in intestinal stem cells [23] (Figure S1f, Table S1).

Collectively, these results demonstrate an association between tumor cell differentiation and metabolic identities in this three-dimensional in vitro CRC model.

### 2.5. Cell States in Patient-Derived CRC Organoids, Xenografts, and Primary Tumors

To assess whether cell types and transcriptional programs identified in tumor spheroids are present in other *LGR5*^+^ CRC models and patient tumors, we analyzed three patient-derived organoids (PDOs; O1–O3), two patient-derived xenografts (PDXs; X1, X2), and cells from three primary tumor samples (T1–T3) by droplet-based scRNA-seq [37,38] (Table 1). 3003–8785 cells passed quality control per PDO. A mean of 4176–5542 genes per cell were detected. For the PDXs, 1475 (X1) and 1070 cells (X2) passed quality control, with a mean of 1841 and 4598 detected human genes per cell, respectively (Table 2).

To distinguish functionally distinct subpopulations, *LGR5* levels were determined and sufficient levels detected in O1, O3, and X1. Since the absolute cell numbers after quality control in the primary tumors were low (T1: 362; T2: 538; T3: 724 cells), the three primary samples were merged and analysis focused on epithelial cells only (total: 253 cells). As observed in spheroids, clustering of cells from *LGR5*^+^ PDO, PDX, and primary tumor cells revealed subpopulations of stem-like, TA-like, or Tdiff-like cells. Additionally, a prominent fraction of Paneth-like (deep crypt secretory-like, *REG4*^+^) cells [39] was detected in the PDX (Figure S3a–c).

Importantly, applying the signatures identified by NNMF of spheroid scRNA-seq data (Table S1) revealed similar trends for heterogeneous metabolic states associated with distinct cell types, that is, OXPHOS in stem-like and TA-like, and glycolysis/hypoxia in Tdiff-like cells (Figure 3b–d). This shows that transcriptional states and cellular composition identified in spheroids are representative of further patient-derived CRC models as well as patient tumors.

### 2.6. Spatial Distribution of OXPHOS and Distinct Cell Types in CRC Spheroids

To analyze spatial organization of metabolic states, we stained spheroids with mitochondrial live-dyes for visualization of mitochondrial membrane potential (MMP) and OXPHOS activity. Histological examination of spheroids (*n* = 3 cultures) revealed crypt-like structures formed by partially polarized cells around lumina, morphologically showing some degree of differentiation. Cells within individual spheroids demonstrated highly heterogeneous MMP, with MMP^high^ cells consistently localized at outer ‘budding’ regions of spheroids and around crypt-like structures (Figure S4a).

**Figure 4:**
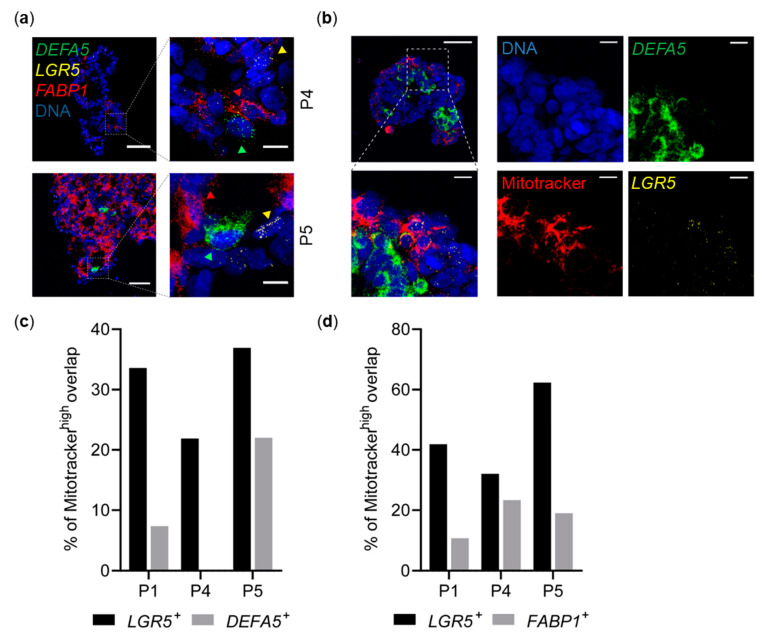
Spatial distribution of metabolic activity and distinct cell types in individual colorectal cancer (CRC) spheroids. (**a**) Histological sections of CRC spheroids co-stained for representative lineage-specific marker genes by RNA fluorescence in situ hybridization (FISH). Left: Overview images. Scale bar, 50 μm. Right: Magnified images representing dashed box regions in overview images (4× digital zoom). Scale bar, 10 μm. *DEFA5*: Paneth-like, *FABP1*: terminally differentiated-like, *LGR5*: stem-like cells. Colored arrowheads mark associated subtypes in magnified images. Images represent z projections from 10 μm slices and DNA is counterstained by 6′-diamidino-2-phenylindole (DAPI). (**b**) Histological section of a spheroid (P1) stained for cell type-specific marker genes (RNA-FISH) and mitochondria (Mitotracker). Top left: Merged overview image. Scale bar, 50 µm. Bottom left: Magnification of dashed region in top left image (4× digital zoom). Scale bar, 10 µm. Center and right: Single channels. Scale bar, 10 µm. Images represent z projections from 10 μm slices and DNA is counterstained by DAPI. (**c**,**d**) Fraction of Mitotracker ‘ON’ cells as determined by automated image analysis pipeline. (**c**) *LGR5*^+^ or *DEFA5*^+^ cells (total number of cells analyzed: P1: *n* = 7379; P4: *n* = 2670; P5: *n* = 2213). (**d**) *LGR5*^+^ or *FABP1*^+^ cells (total number of cells analyzed: P1: *n* = 3403; P4: *n* = 1580; P5: *n* = 1601).

Multiplexed RNA fluorescence in situ hybridization (FISH) for intestinal cell type markers *LGR5* (stem-like), *DEFA5* (Paneth-like), and *FABP1* (Tdiff-like) resulted in discrete staining of individual cells by either a single or none of the markers, indicating existence of distinct intestinal cell types in all three patient cultures. Cellular subtypes also showed tendencies for spatial localization. *DEFA5*^+^ cells were primarily detectable in inner regions of spheroids. *LGR5*^+^ cells preferably localized towards outer regions. Frequently, *DEFA5*^+^ cells were identified in proximity to *LGR5*^+^ cells (Figure 4a,b). In the intestinal crypt, *LGR5*^+^ cells reside at the crypt base [27,40], and—in line with our observations—imaging of intestinal organoids has shown localization of *LGR5*^+^ cells close to Paneth cells [41].

To correlate MMP with specific cell types, we combined mitochondrial staining and multiplexed RNA-FISH, showing *DEFA5*^+^ and *FABP1^+^* cells to be largely excluded from MMP^high^ regions, whereas *LGR5*^+^ cells were primarily located in MMP^high^ regions. Matching our scRNA-seq results, quantitative image analysis in thousands of single cells revealed that the fraction of *LGR5*^+^ cells located in MMP^high^ regions is indeed much higher compared to *DEFA5*^+^ and *FABP1*^+^ cells in all examined cultures (*n* = 3; Figure 4c,d, Figure S4b,c).

Hence, in situ RNA fluorescence microscopy further confirmed cell type-specific metabolic preferences of putative stem-like, Paneth-like, and Tdiff-like cell subtypes in CRC. In addition, metabolic activities of cellular subtypes are associated with specific spatial localization within spheroids.

### 2.7. Heterogeneous Energy Metabolism in Patient Tumors

Identification of cell type-specific metabolic preferences in patient-derived CRC cultures raised the question whether heterogeneously expressed metabolic signatures can be identified directly in CRC patient tumors. To address this, we analyzed primary tumors (*n* = 25 patients) and liver metastases (*n* = 25 patients) by immunohistochemistry for expression of LDH-A and CA9 (Figure S5a,b) as marker genes of hypoxia/glycolysis and Tdiff signatures (Figure S1f, Table S1).

Within the majority of examined specimens, immunohistochemical analysis revealed that only subfractions of all cells express LDH-A and CA9, indicating existence of metabolic heterogeneity within individual patient tumors. Despite high expression of the proliferation marker MKI67, previously reported to preferentially mark TA-like cells [42], regions of CA9 expression were largely overlapping with MKI67^−^ areas in most patient tumors, suggesting that tumor cells with expression of the hypoxia/glycolysis signature were indeed less proliferative, and actively cycling TA-like cells might prefer OXPHOS to generate energy (Figure S5c).

### 2.8. Heterogeneous Energy Metabolism in Patient-Derived Models

**Figure 5:**
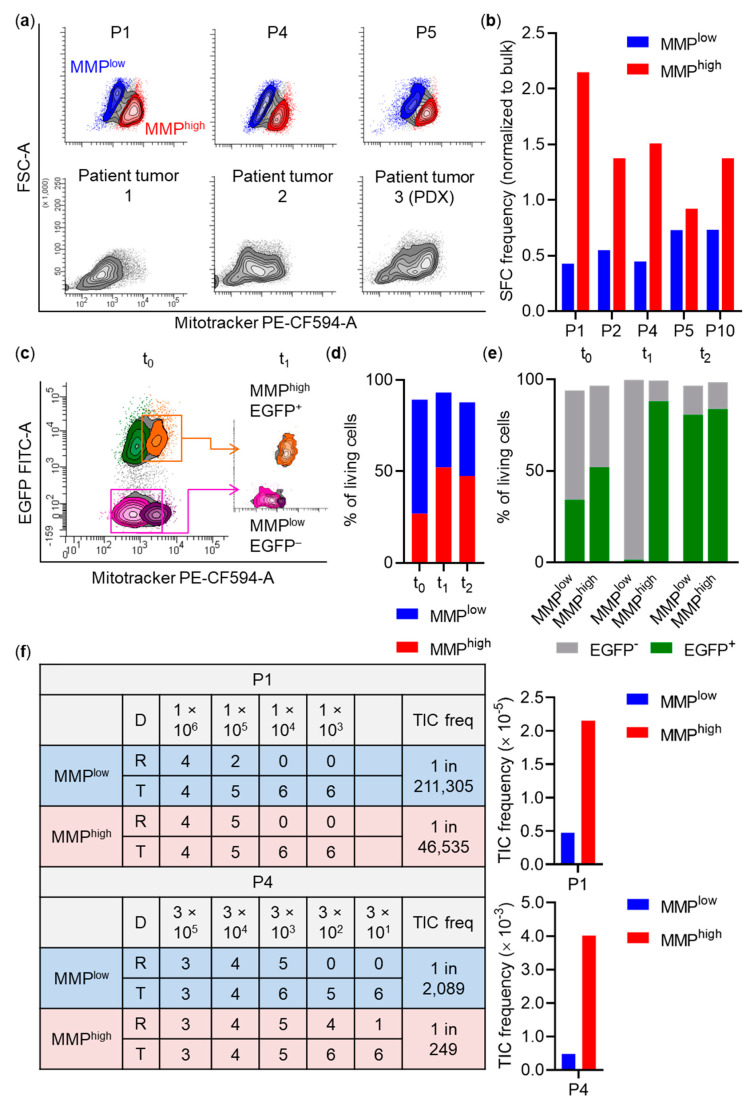
Association of tumor-initiating cell (TIC) activity and mitochondrial membrane potential (MMP). (**a**) Top: Heterogeneous MMP staining (Mitotracker) of spheroid cells assessed by flow cytometry (representative plots shown). Colored cell populations indicate sorted fractions. Bottom: Heterogeneous MMP staining pattern in tumor cells. Axis scale numbers are representative for all plots. PDX, patient-derived xenograft. (**b**) Spheroid-forming cell (SFC) frequencies of MMP sorted spheroid cells (P1, P4: *n* = 3; P5: *n* = 2; P2, P10: *n* = 1) determined by in vitro limiting dilutions. SFC frequencies were calculated based on sphere formation 5–7 days after seeding and normalized to bulk (P1, P4, P5: *n* = 2; P2, P10: *n* = 1). (**c**) Experimental layout of co-cultivation experiments. x- and y-axis are displayed biexponentially. Results for a representative spheroid culture (P1) are shown. Axis scale numbers are representative for all plots. EGFP, enhanced green fluorescent protein. (**d**,**e**) Composition of (**d**) MMP^low^ and MMP^high^ cells or (**e**) EGFP^–^ and EGFP^+^ cells in the MMP^low^ and MMP^high^ subpopulations over time. t_0_: before, t_1_: directly after, t_2_: 21 days after sort. (**f**) Results of limiting dilutions in vivo. Left: Overview of dose (D) and response (R). Right: TIC frequencies of MMP sorted spheroid cells. TIC frequencies were calculated based on tumor formation seven weeks (P1: *n* = 42 mice) or five weeks (P4: *n* = 48 mice) after transplantation. Freq, frequency; T, tested.

To assess whether cellular subfractions with distinct OXPHOS activities can be identified in viable cells, we used an MMP dye for flow cytometry allowing distinction of cells with different mitochondrial activity based on fluorescence intensity. Indeed, heterogeneous fluorescence intensities allowed separation of populations with different MMP (MMP^low^, MMP^high^; Figure 5a).

To understand whether heterogeneous metabolic activity is relevant in patient CRC tumors, we determined OXPHOS activity of two freshly purified patient tumors and a patient tumor expanded as PDX in vivo by flow cytometry-based MMP analysis. In all samples, two cell populations with distinct MMP were identified (Figure 5a), indicating that heterogeneous mitochondrial activity also exists in PDXs and patient tumors.

This finding was further supported by proteomic analysis of MMP^low^ and MMP^high^ populations of *LGR5*^+^ (i.e., *LGR5* score > 1) spheroid cultures (P1, P4, P7, P11) which revealed differentially abundant proteins between the two populations. Interestingly, three proteins contributing to the stem-like signature (PROX1, GRN, DEFA6; Table S1) were significantly higher abundant in MMP^high^ compared to MMP^low^ (Figure S5d).

### 2.9. Increased Spheroid and Tumor Formation Capacity in OXPHOS^High^ Cells

scRNA-seq data suggested that subfractions of MMP^low^ and MMP^high^ spheroid cells preferentially harbor Tdiff-like and Paneth-like (MMP^low^) or stem-like and TA-like tumor cells (MMP^high^). As spheroid and tumor forming capacity is supposed to be restricted to stem-like tumor cells [43], we calculated spheroid-forming cell (SFC) frequencies in vitro and TIC frequencies in vivo by limiting dilutions of sorted MMP^low^ and MMP^high^ cell fractions.

SFC frequencies were strongly increased in MMP^high^ cell fractions compared to MMP^low^ fractions or bulk spheroid cells in four out of five cultures (Figure 5b). Spheroid cells (P1) sorted according to JC-1 aggregation—a different MMP indicator—also demonstrated increased SFC frequency in the MMP^high^ subpopulation (MMP^high^: 1/26; MMP^low^: 1/46; Figure S5e).

As increased mitochondrial OXPHOS is linked to enhanced ROS levels [23], we further assessed the association of SFC frequency and OXPHOS by staining spheroid cells (P4) with a live-dye fluorescent upon ROS oxidation. In vitro limiting dilutions revealed substantial enrichment of SFCs in the sorted ROS^high^ compared to the ROS^low^ subpopulation (ROS^high^: 1/9; ROS^low^: 1/117; Figure S5f).

We then asked whether MMP^high^ cells exhibit a growth advantage in competition with MMP^low^ cells. Spheroid cultures (P1, P4, P5) were transduced with a lentiviral vector encoding for enhanced green fluorescent protein (EGFP) under the control of the human phosphoglycerate kinase (PGK) promoter in order to allow follow up of sorted populations by assigning presence or absence of EGFP expression to the metabolic state at the time of sort. To achieve this, ~40–50% EGFP^+^ cultures were stained for MMP (t_0_) and cells were sorted as co-cultures of MMP^high^EGFP^+^ and MMP^low^EGFP^–^ spheroid cells (1:1 ratio; t_1_). After three weeks (t_2_), despite similar relative contributions of MMP^low^ and MMP^high^ fractions, co-cultures were nearly completely EGFP^+^, indicating a growth advantage of MMP^high^ compared to MMP^low^ cells (Figure 5c–e).

To quantify TIC frequency in MMP^high^ and MMP^low^ subpopulations in vivo, spheroid cultures (*n* = 2) were sorted according to MMP. Descending cell numbers of each population were subcutaneously injected into NSG mice. For P1, 42 mice with four different dilutions (10^3^–10^6^ cells), for P4, 48 mice with five different dilutions (3 × 10^1^–3 × 10^5^ cells) were transplanted. For all mice where endpoint criteria have not been reached before, tumor formation was assessed simultaneously at defined endpoints (P1: seven weeks; P4: five weeks after transplantation). Importantly, in both tested cultures, calculated TIC frequencies were substantially increased in MMP^high^ compared to MMP^low^ cells (P1: 1/46,535 vs. 1/211,305; P4: 1/249 vs. 1/2089 for MMP^high^ vs. MMP^low^, respectively; Figure 5f), demonstrating strong enrichment of stem-like tumor cells in the MMP^high^ population.

Cell type-specific metabolic preferences might represent a targetable metabolic vulnerability in CRC. To test this hypothesis, we assessed the impact of carbonyl cyanide *m*-chlorophenyl hydrazine (CCCP), a drug perturbing adenosine triphosphate synthesis by transporting protons across the mitochondrial inner membrane [44], on SFC frequency (*n* = 3 spheroid cultures). Upon 4 h pretreatment with 25 µM CCCP, a lower SFC frequency of CCCP compared to dimethyl sulfoxide (DMSO) treated cells was observed for all cultures tested (P1: 1/19 vs. 1/14; P4: 1/18 vs. 1/9; P5: 1/68 vs. 1/14 for CCCP vs. DMSO treated, respectively; Figure S5g), indicating sensitivity of stem-like cells towards OXPHOS inhibition.

## 3. Discussion

We here analyzed functional CRC intra-tumor heterogeneity at single-cell level and demonstrate that distinct functional programs within individual CRC cells can be assigned to specific cellular subpopulations.

In healthy tissues including normal intestine, functional cellular heterogeneity is established by differentiation processes of stem and progenitor cell populations, which control the tissues’ functionality in a demand-dependent manner [27]. Similarly, in CRC and other solid tumors as well as in hematological malignancies, functional heterogeneity of tumor and non-tumor cells in the surrounding microenvironment exists and acts as driver of tumor progression [31,45]. Although the majority of tumor cells in CRC cycles actively, we identified proliferatively inactive cells in patient-derived cultures and within established xenograft tumors in vivo—in line with recent data on the healthy intestine [46]. Nevertheless, these cells eventually can re-enter the cell cycle and exhibit TIC activity, suggesting that cells escape from a quiescent state, possibly driven by cellular plasticity as described before for CRC [47]. Accordingly, slow or non-cycling cells were suggested to exhibit increased chemoresistance and drive relapse following initial successful therapy [17,48].

This has striking parallels to the normal intestine, where ablation of stem cells under pathological conditions (e.g., irradiation) can be compensated by a reserve pool of stem cells that are rare during homeostasis but can regenerate all different cell populations including stem, progenitor, and differentiated cell types upon activation, thereby maintaining a functional intestine after tissue injury [49].

The complex composition of different subpopulations within normal and malignant intestinal epithelium and their dynamic interactions are poorly understood. Their characterization has been hampered by the dependency of experimental approaches on purifying cell populations, which cannot fully distinguish between or comprehensively capture distinct cell types and intermediates and might fail to detect rare and poorly characterized cell populations. Recent studies shed light on this complexity by utilizing single-cell approaches to detect and characterize rare cell types in the normal intestine and CRC [42,46,49,50,51,52,53]. We here demonstrate that scRNA-seq further allows the identification of cell type-specific expression modules in CRC and enables identification of functional states during TIC differentiation based on transcriptional heterogeneity.

In line with observations in other entities, transcriptional programs across multiple CRC patients were dominated by inter-patient heterogeneity, most likely due to individual genetic and epigenetic alterations [21,25,54]. Interestingly, most patient-derived spheroid cells clustered according to primary tumor or metastasis site, suggesting either a stable effect of tumor environment on transcriptional programs or selection of tumor cells with specific expression profiles.

Gene sets most heterogeneously expressed within individual spheroids and PDOs included genes specifically expressed in distinct cell types of the normal intestinal epithelium (e.g., a gene set including *LGR5* for stem-like, a gene set including *KRT20* for Tdiff-like cells) [55]. This further supports the notion that, in CRC, there exist functionally distinct cell types that phenotypically reflect those of the normal intestinal epithelium [6,8]. Still, in contrast to the normal intestinal epithelium where distinct cellular subpopulations can be discriminated in high resolution by scRNA-seq technologies [55], gene expression within identified subfractions of CRC was less distinct. However, individual subfractions shared transcriptional traits, potentially reflecting continuous cell type transitions after malignant transformation comparable to reports on hematopoietic stem cell differentiation [56]. In glioblastoma, bulk RNA-sequencing of individual tumors was used to analyze transcriptional heterogeneity and identified different tumor subtypes, while scRNA-seq revealed different proportions of cell types within individual tumors underlying transcriptional heterogeneity rather than distinct homogeneous tumor subtypes [54]. This is in line with our data showing cellular diversity of cell types and cell states within individual patient tumors. Of note, four out of 12 spheroid cultures did not meet inclusion criteria for NNMF analysis due to low *LGR5* scores. Accordingly, previous findings show that, while *LGR5*^+^ tumor cells can be detected in tumors from all CRC subtypes independent of their cellular composition [53], up to a third of individual CRCs tumors may lack detectable *LGR5* levels [14]. Furthermore, *LGR5* plasticity has recently been shown to drive CRC metastasis [57]. In the presented study, we only focused on patient-derived cultures with high expression of *LGR5*. Future analyses of the hierarchical organization of *LGR5*^–^ cultures and existing cellular subpopulations in comparison to the cellular subpopulations and cellular states described in this study could further widen the understanding of cellular heterogeneity in CRC.

Our approach to decipher transcriptional programs heterogeneously expressed in functionally distinct CRC cell subfractions identified heterogeneous gene expression programs related to cell cycle, immune response, and metabolic states like OXPHOS and glycolysis. Given the considerable functional and proliferative differences between distinct cell populations, cell-to-cell variability in energy turnover and demand appears likely. A recent study has linked decreased biosynthetic capacities to differentiation [58]. As OXPHOS can be more efficient in energy production [59], highly proliferative TA-like tumor cells might prefer OXPHOS over glycolysis to generate energy. Even though such cell type-specific metabolic identities are known from the normal intestinal epithelium [23], distinct metabolic preferences within normal and malignant stem cell systems are not uniform across different tissue types and tumor entities, and are not necessarily correlated with proliferation activity in general. For example, TICs in hepatocellular carcinoma [60], breast cancer [61], osteosarcoma [62], and nasopharyngeal carcinoma [63] rely on glycolysis for tumor formation, while TICs in pancreatic ductal adenocarcinoma [64], glioma [65], and acute myeloid leukemia [66] prefer OXPHOS. Importantly, tumor cells can also alternate between glycolysis and OXPHOS, thereby adapting to metabolic challenges [67].

Here, we were able to assign the metabolic demand of OXPHOS to functionally relevant stem-like and TA-like cells and observed substantial enrichment of self-renewing and proliferating SFCs and TICs in OXPHOS^high^ cell subfractions. As a consequence, inhibition of OXPHOS impaired spheroid formation in vitro identifying OXPHOS as a novel druggable target in CRC. Since high OXPHOS levels were detected in stem-like and TA-like cell compartments, targeting OXPHOS as treatment strategy might eliminate the most self-renewing and proliferating cell types simultaneously.

Interestingly, stem-like tumor cells demonstrated overexpression of *OXR1* and *PON2*, both involved in protection against ROS accumulating as co-product of OXPHOS [34]. Further studies are needed to address whether expression of *OXR1* and *PON2* may be involved in a mechanism by which this long-lived and thus vulnerable population of stem-like tumor cells protects itself against ROS-mediated damage.

In our proteomic analysis, proteins significantly higher abundant in the MMP^high^ subpopulation included PROX1, one of the top markers of the stem signature and usually expressed in the enteroendocrine lineage [51]. Interestingly, *PROX1* has been reported to be positively correlated with *LGR5* expression in CRC [43] and linked to stem cell maintenance and metastasis [68,69]. Another protein significantly more abundant in MMP^high^ was DEFA6, a protein expressed in normal Paneth and Paneth-like tumor cells [70]. Its moderate expression in the stem-like cell population might reflect a continuous rather than a stepwise process underlying transition from stem-like to Paneth-like cell subsets (and potentially vice versa) in CRC. While Paneth cells constitute the niche for LGR5^+^ cells in the small intestinal epithelium, this function is performed by REG4-expressing deep crypt secretory cells in the colon [39,71,72]. *REG4* was also included in the NNMF Paneth-like signature, suggesting that both cell types might contribute to this signature.

Of note, the expression signatures identified by scRNA-seq of patient-derived CRC spheroids have shown a prognostic relevance for CRC patients comparable to previously reported subtypes linked to cancer-associated fibroblasts [31] or CMSs [29], indicating that cell types and cell states might indeed be biologically distinct and of potential clinical relevance for CRC patients.

In summary, we here show that distinct functional cell states during TIC differentiation can be identified by single-cell transcriptomes. Targeting differentiation of cancer cells and associated transcriptional states might represent a novel therapeutic strategy for human CRC.

## 4. Materials and Methods

### 4.1. Primary CRC Spheroids and Organoids

Human CRC samples (male and female patients) were obtained from Heidelberg University Hospital in accordance with the Declaration of Helsinki. Informed consent on tissue collection was received from each patient, as approved by the University Ethics Review Board on 19 May 2009 (323/2004) and 7 June 2013 (S-649/2012). Tumor sample processing and purification procedures were described previously [4,73,74].

For generation of three-dimensional spheroid cultures, cells freshly isolated from patient material or PDXs were cultivated in ultra-low attachment flasks (Corning, Corning, NY, USA) in serum-free culture medium (Advanced DMEM/F-12 supplemented with 0.6% glucose, 2 mM L-glutamine (all ThermoFisher, Waltham, MA, USA), 5 mM HEPES, 4 µg/mL heparin (all Sigma-Aldrich, St. Louis, MO, USA), 4 mg/mL bovine serum albumin (PAN-Biotech, Aidenbach, Germany)). Growth factors (20 ng/mL epidermal growth factor, 10 ng/mL fibroblast growth factor basic (all R&D Systems, Minneapolis, MN, USA)) were added twice per week.

To dissociate tumor spheroids, cells were pelleted, resuspended in 0.25% trypsin-EDTA (ThermoFisher, Waltham, MA, USA), and incubated for 10–30 min at 37 °C. The reaction was stopped by adding 20% fetal bovine serum (PAN-Biotech, Aidenbach, Germany) in phosphate-buffered saline (PBS; ThermoFisher, Waltham, MA, USA). Cells were pelleted, resuspended in medium, and filtered through a 40 µm cell strainer (Corning, Corning, NY, USA). To avoid secondary cell culture artifacts, like hypoxic cores in large spheroids [75], cultures were dissociated at defined, pretested time points 6–14 days before individual experiments.

For generation of three-dimensional organoid cultures, purified cells were seeded in 10 µL drops of Cultrex reduced growth factor basement membrane extract (R&D Systems, Minneapolis, MN, USA) into not-treated 6-well plates (Corning, Corning, NY, USA). Organoids were cultured as previously described with minor modifications [76,77] and in the absence of WNT, R-spondin and Noggin, thereby selecting for tumor cells with activation of WNT/β-catenin signaling and inhibition of BMP signals [78,79]. In brief, cells were cultured in serum-free culture medium (Advanced DMEM/F-12 supplemented with B-27 supplement, 2 mM L-glutamine, 100 µg/mL streptomycin, 100 U/mL penicillin (all ThermoFisher, Waltham, MA, USA), 10 mM HEPES, 10 mM nicotinamide, 1.25 mM *N*-acetyl-L-cysteine, 1 µM SB 202190, 500 nM A 83-01, 10 nM gastrin, 10 nM prostaglandin E_2_ (all Sigma-Aldrich, St. Louis, MO, USA), 100 µg/mL primocin (InvivoGen, San Diego, CA, USA)). 20 ng/mL of epidermal growth factor was added three times per week and medium was exchanged weekly. After seeding, 10 µM Y-27632 (StemCell Technologies, Vancouver, BC, Canada) was added. To dissociate tumor organoids, cells were taken up in 0.25% trypsin-EDTA diluted 1:1 in PBS and incubated for 10–20 min at 37 °C. To enhance dissociation, organoids were mechanically disrupted by pipetting. The reaction was stopped by adding 20% fetal bovine serum in PBS. Cells were washed twice with PBS before reseeding.

Spheroid and organoid cultures were authenticated using Multiplex Cell Authentication by Multiplexion (Heidelberg, Germany) as described [80]. The SNP profiles matched known profiles or were unique. The purity of spheroid and organoid cultures was validated using the multiplex cell contamination test by Multiplexion (Heidelberg, Germany) as described recently [81]. No mycoplasma, SMRV or interspecies contamination was detected. To assure pure epithelial cell content and exclude contaminations with murine or hematopoietic cells, established cultures were tested for EPCAM, H2kd, and CD45 expression by flow cytometry.

### 4.2. Laboratory Animals

Male and female immunodeficient NSG mice purchased from The Jackson Laboratory (Bar Harbor, ME, USA) were further expanded in the Centralized Laboratory Animal Facilities of the DKFZ, Heidelberg. Animals were group-housed in standard individually ventilated cages with wood chip embedding (LTE E-001, ABEDD, Vienna, Austria), nesting material, autoclaved tap water and ad libitum diet (autoclaved mouse/rat housing diet 3437, Provimi Kliba, Kaiseraugst, Switzerland). Room temperature and relative humidity were adjusted to 22.0 ± 2.0 °C and 55.0 ± 10.0%, respectively, in accordance with Appendix A of the European Convention for the Protection of Vertebrate Animals used for Experimental and Other Scientific Purposes from 19 March 1986. According to FELASA recommendations, all animals were housed under strict specific pathogen-free conditions. The light/dark cycle was adjusted to 14 h lights on and 10 h lights off with the beginning of the light and dark period set at 6 am and 8 pm, respectively. The age of transplanted mice was at least seven weeks. All animal experimentation performed in this study was conducted according to national guidelines and was reviewed and confirmed by an institutional review board/ethics committee headed by the responsible animal welfare officer. The Regional Authority of Karlsruhe, Germany finally approved the animal experiments as the responsible national authority (approval numbers G228/12 (29 January 2013), G49/14 (26 June 2014), G233/15 (17 November 2015)).

### 4.3. scRNA-seq of Spheroids

To generate single-cell suspensions, cells were trypsinized as described. Trypsinization was enhanced by applying shear forces with a pipette every 5 min. After stopping the reaction, cells were washed twice with PBS and filtered through a 15–20 µm cell strainer (PluriSelect, Leipzig, Germany). To count and test for cell viability using an automated cell counter (Countess, ThermoFisher, Waltham, MA, USA), single-cell suspensions were stained with Hoechst and propidium iodide (ReadyProbes Cell Viability Imaging Kit, ThermoFisher, Waltham, MA, USA) for 10 min at room temperature. Only samples with at least 85% viability were used for further processing. For isolation of single cells, reverse transcription, and cDNA amplification, the Rapid Development Kit (Wafergen, Fremont, CA, USA; compare: SMARTer iCELL8 3′ DE Reagent Kit, TakaraBio, Kusatsu, Japan) for in-chip reverse transcription-PCR amplification with the iCELL8 system (TakaraBio, Kusatsu, Japan) [19] was used. The cell suspension was diluted to 25 cells/µL. Cells were dispensed from a 384-well source plate into a nanowell chip (SmartChip v1/v2 kit, TakaraBio, Kusatsu, Japan; P7: v2; others: v1) containing uniquely barcoded oligo-dT primers for each well, resulting in up to 30% of wells containing single cells following Poisson distribution. Wells were imaged using an automated fluorescence microscope (BX43, Olympus, Shinjuku, Japan) and image processing was performed using CellSelect (TakaraBio, Kusatsu, Japan). Additional manual curation for multiplets and dead cells was performed. 50 nL RT/Amp solution was dispensed into nanowells (master mix: 56 μL 5 M betaine (Sigma-Aldrich, St. Louis, MO, USA), 24 μL 25 mM dNTP mix (TakaraBio, Kusatsu, Japan), 3.2 μL 1 M magnesium chloride (ThermoFisher, Waltham, MA, USA), 8.8 μL 100 mM dithiothreitol, 61.9 μL 5× SMARTScribe first-strand buffer, 33.3 μL 2× SeqAmp PCR buffer, 4.0 μL 100 μM RT E5 oligo, 8.8 μL 10 μM Amp primer (all TakaraBio, Kusatsu, Japan), 1.6 μL 100% Triton X-100 (ThermoFisher, Waltham, MA, USA), 28.8 μL SMARTScribe reverse transcriptase, 9.6 μL SeqAmp DNA polymerase (all TakaraBio, Kusatsu, Japan)). In-chip RT/Amp amplification was performed for 18 amplification cycles (Bio-Rad, Hercules, CA, USA; modified for iCELL8 chips). Libraries were pooled, concentrated (DNA Clean&Concentrator-5, Zymo Research, Irvine, CA, USA), purified (0.6× Ampure XP beads, Beckman Coulter, Brea, CA, USA), and assessed for DNA quality (Bioanalyzer and High Sensitivity DNA Kit, Agilent, Santa Clara, CA, USA). Next generation sequencing libraries were constructed following manufacturer’s instructions using the Nextera XT DNA Library Prep Kit (Illumina, San Diego, CA, USA) and sequenced using NextSeq500 (Illumina, San Diego, CA, USA; high-output mode, paired-end; v1 chip: 21 × 70 bp; v2 chip: 24 × 67 bp).

### 4.4. scRNA-seq of Tumors, PDXs, PDOs

To generate single-cell suspensions, cells were trypsinized as described. After stopping the reaction, cells were washed with PBS and filtered through a 40 µm cell strainer. Cells were washed, resuspended in PBS supplemented with 0.05% bovine serum albumin, and filtered through a 20 µm cell strainer. Single-cell suspensions were loaded following the Chromium Single Cell 3′ Library Kit v2 (10× Genomics, Pleasanton, CA, USA) protocol to generate cell and gel bead emulsions. Reverse transcription, cDNA amplification, and sequencing library generation were performed according to manufacturer’s protocol. Each library was sequenced in one lane of the NextSeq500 (Illumina, San Diego, CA, USA; high-output mode, paired-end, 26 × 49 bp).

### 4.5. Preprocessing and Analysis of iCELL8 Data

scRNA-seq data were preprocessed using an automated in-house workflow (Roddy; https://github.com/TheRoddyWMS/Roddy). FastQC was used to evaluate read quality. Assignment of iCELL8 library barcodes to corresponding nanowells was performed with the Je demultiplexing suite [82]. Sequences were trimmed for primer sequences, poly-A/T tails, and low-quality ends using Cutadapt with the ‘--nextseq-trim’ option. Mapping to the reference genome hs37d5 was performed (STAR aligner). Quantification of mapped BAM files was performed using featureCounts (reference annotation gencode v19). Only scRNA-seq libraries matching the following criteria were used: (i) >100,000 reads, (ii) >1000 detected genes, (iii) <15% mitochondrial reads. Strong PCA outliers as well as libraries with top 5% of reads for every patient independently were removed. As previously published [25], expression levels based on raw read counts were quantified as

(1) $$E_{i,j}=\log_{2}\left(\frac{CPM_{i,j}}{10}+1\right)\;,$$

with *CPM_i,j_* as the counts-per-million for gene *i* in sample *j*. Aggregate expression of each gene across all cells was calculated as

(2) $$E_{a}=E_{i,j}=\log_{2}\left(mean\left[E_{i,1\ldots n}\right]+1\right)$$

with genes with *Ea* < 3.5 being excluded to focus on highly or intermediately expressed genes.

Combined filtered and normalized data of all patients were used for evaluation of inter-patient gene expression differences. The R package Seurat [38] was used for identification of highly variable genes, PCA, clustering, two-dimensional visualization, and differential expression analysis (Wilcoxon rank sum test: adjusted *p*-value < 0.05; log fold-change > 0.25).

Before combining the data of all patients, relative expression levels were calculated individually for each patient using a mean-centering approach

(3) $$Er_{i,j}=E_{i,j}-mean\left[E_{i,1\ldots n}\right]$$

to eliminate global inter-patient gene expression shifts.

PCA was applied and—for visualization—the top 30 genes with low and high scores in the first principal component were clustered using average group linkage (UPGMA) by the ‘aheatmap’ function from R’s ‘NMF’ package. Gene set enrichment analysis [83] was performed on the top 300 genes with highest and lowest PC scores.

Transcriptional signatures shared across patients were identified using NNMF [24] of mean-centered data of all patients defined as *LGR5*^+^ (*n* = 8 patients; Table 2). Analysis was performed in MATLAB (MathWorks, Natick, MA, USA; ‘nnmf’) with a factor number of *k* = 25 and negative events set to 0. To exclude patient-specific signatures, pairwise overlaps in frequency distributions of cell scores for individual factors were determined and factors with overlaps <50% in at least five patients were excluded. Biological relevance of factors and their associated genes was analyzed manually and by gene set enrichment analysis [83]. Factors potentially driven by technical artifacts were excluded. Signature scores were defined as averaged expression of the top 200 genes per factor. To reduce redundancy for visualization, signatures showing similar enrichment and clustering patterns were combined to meta-signatures (Figure S1a–e, Table S1).

Meta-signature scores (calculated based on the combined gene lists of the comprised signatures) were clustered using complete linkage of Euclidean distances. NNMF analysis was repeated with various numbers of factors resulting in identification of similar core signatures.

To test whether cell type-specific transcriptional programs (stem-like, TA-like, Paneth-like, Tdiff-like) are active in individual cells or—in other words—to differentiate between cells that belong to the four cell type-specific subpopulations, we adapted the above described cell scoring approach based on the expression of inferred NNMF meta-signatures [25] and used control random gene sets as background model to control for technical confounders as library complexity. Cell type-specific transcriptional programs were defined as active if their expression in individual cells was >1 standard deviation above the mean across all cells. Inferred cell state-specific signatures were scored for cells of a particular cell type to assess the degree to which cell states are active in specific cell types.

### 4.6. Preprocessing and Analysis of 10x Data

For 10× 3′ libraries generated from cells derived from PDOs, PDXs, and primary tumor samples, raw sequencing data were processed using CellRanger (10× Genomics, Pleasanton, CA, USA; version 2.1.1). Transcripts were aligned with the 10× reference human genome hg19 1.2.0 and the mouse genome mm10 1.2.0. Quality control and downstream analysis were performed with Seurat (https://github.com/satijalab/seurat; version 3.0.0). Only cells matching the following criteria were used for downstream analysis: PDOs: (i) >2000 detected genes, (ii) <10% mitochondrial reads; PDXs: (i) >500 detected genes, (ii) <10% mitochondrial reads for *Homo sapiens*, and (i) >1000 and <4500 detected genes, (ii) <10% mitochondrial reads for *Mus musculus*; primary tumor samples: (i) >200 and <6000 detected genes, (ii) <15% mitochondrial reads. Only human cells from the PDXs and epithelial cells (*EPCAM*^+^, *VIL1*^+^, *CEACAM5*^+^, *VIM*^–^, *SPARC*^–^) from the primary tumor samples were analyzed.

Subsequent downstream analysis was performed with standard Seurat workflow, including log-normalization and scaling as well as PCA and clustering using the top 2000 variable genes. Datasets were visualized using two-dimensional t-distributed stochastic neighbor embedding maps [84]. The three primary tumor samples were aligned using canonical correlation analysis implemented in Seurat [85]. In brief, this method identifies pairwise correspondences between single cells across different datasets belonging to specific biological states, termed ‘anchors’. These anchors are the basis of harmonizing datasets. Differentially expressed genes between identified clusters were identified using Wilcoxon rank sum test. Identified clusters were scored for cell state signatures using the ‘AddModuleScore’ function (Seurat), using gene signatures from NNMF analysis (Table S1).

### 4.7. Patient Clustering and Survival Analysis

Cell type and cell state signatures obtained from spheroid scRNA-seq data (Table S1) were evaluated in a patient survival analysis. Bulk transcriptomic data for COAD patients with available survival data were collected from TCGA (level 3 RNA-seq, *n* = 328 patients) [28] and log-transformed. For each TCGA patient, the mean expression of gene signatures was calculated and used to cluster bulk transcriptomes by complete linkage of Euclidean distances. Patients were grouped according to different combinations of cell type and cell state signatures. In a new clustering process, the sample space was progressively subdivided using the main signatures defining each cluster of patients: First, OXPHOS_1, G1/S, G2/M, and stem signatures separate cl2 and cl3 (high) from the rest (low; Euclidean distances). Then, hypoxia/glycolysis_1 and TNFα_2 signatures distinguish cl2 (low) from cl3 (high; Euclidean distances). Next, fatty acid and TNFα_1 signatures separate cl1 (high) from cl4, cl5, and cl6 (Euclidean distances). Subsequently, stem and TA signatures separate cl6 (stem^low^) from cl4 and cl5 (stem^high^; correlation). Finally, G1/S, G2/M, and OXPHOS_1 signatures also distinguish between cl4 (medium) and cl5 (low; Euclidean distances). Complete linkage of Euclidean distances was used to cluster stem^high^ and stem^low^ patients. Kaplan–Meier survival curves were generated using ‘survival’ and ‘survminer’ libraries in R. We performed Cox proportional hazards modeling and multivariable models with and without cell type and cell state clusters were compared by performing analysis of variance (ANOVA). ‘CMScaller’ [29] was used to stratify the TCGA COAD cohort. To generate the contingency table, patients that could not be assigned to a CMS (*n* = 19 patients) were excluded.

### 4.8. Genetic Labelling of Spheroids

For tracking of cells within tumors, lentiviral vector particles encoding for tetracycline-regulated (Tet-off) H2B-GFP were produced in HEK293T cells, concentrated by ultracentrifugation, and titrated on HeLa cells as described [4,5]. Patient-derived spheroid cultures (*n* = 7) were transduced with a multiplicity of infection of 1–20 aiming at transduction efficiencies of ~ 40% to avoid multiple vector integrations. Within 24 h after transduction, 4 × 10^5^–1.7 × 10^6^ transduced cells were transplanted under the kidney capsule of NSG mice (*n* = 14, 1–4 mice per spheroid culture) anesthetized by 1.75% isoflurane (Abbott, Chicago, IL, USA) in the breathing air. Mice were checked daily for tumor growth, and starting two weeks prior to tumor harvesting, doxycycline (Genaxxon, Ulm, Germany) was added to the drinking water of tumor-bearing mice to shut down H2B-GFP expression. Mice were sacrificed, xenograft tumors were digested as described [5,73], cells were stained with 200 nM TOTO-3 (ThermoFisher, Waltham, MA, USA) in Hank’s Balanced Salt solution (Sigma-Aldrich, St. Louis, MO, USA) supplemented with 2% fetal bovine serum for dead cell exclusion, and tumor cells were sorted according to GFP expression intensity (AriaII and FACS Diva, Becton Dickinson, Franklin Lakes, NJ, USA). GFP signal was detected in the FITC channel (488 nm laser; 505 LP, 525/50 filter). TOTO-3 signal was detected in the APC channel (633 nm laser; 670/30 filter). Samples were gated for cells (FSC-A vs. SSC-A), singlets (FSC-A vs. FSC-W, SSC-A vs. SSC-W), and living cells (FSC-A vs. APC-A). Populations with high, medium, and low/absent GFP expression were sorted (SSC-A vs. FITC-A), reanalyzed to test for sort efficiency, and serially transplanted into secondary recipient mice (1 × 10^2^–4.5 × 10^4^ cells; *n* = 33 mice). Mice were monitored daily for tumor formation and sacrificed when tumors reached the maximum tolerable size.

### 4.9. RNA-FISH

For combinatory stainings of mitochondrial activity and mRNA, undissociated spheroids were stained for 3 h with 100 nM Mitotracker Red CMXRos solution (ThermoFisher, Waltham, MA, USA). For histological preparation, cells were fixed in 4% formaldehyde (ThermoFisher, Waltham, MA, USA) for 20 min at 4 °C, washed twice with PBS, and incubated in 30% sucrose overnight at 4 °C. Samples were embedded (Richard-Allan Scientific Neg-50 Frozen Section Medium, ThermoFisher, Waltham, MA, USA) and frozen in the gaseous phase of liquid nitrogen. Histological sections (10 µm slices) were prepared on a cryostat (Leica, Wetzlar, Germany) and mounted on Superfrost Plus slides (ThermoFisher, Waltham, MA, USA). For RNA-FISH, the RNAscope Multiplex Fluorescent v2 (Bio-Techne, Minneapolis, MN, USA) was used according to manufacturer’s instructions with probes targeting mRNAs of *LGR5*, *DEFA5*, and *FABP1*. Alexa488, Atto550, or Atto647 were used as fluorescent dyes. Cryosections were stained with 6′-diamidino-2-phenylindole (DAPI) and mounted in SlowFade Gold Antifade solution (ThermoFisher, Waltham, MA, USA). Images were acquired by confocal laser scanning microscopy (SP8, Leica, Wetzlar, Germany) in 15 z stacks (z range: 20 µm).

For quantitative analysis of RNA-FISH/Mitotracker imaging data, we developed a single-cell image analysis pipeline to relate metabolic activity (Mitotracker) to intestinal subtypes (RNA-FISH). To prepare spheroid images for further analysis, we performed maximum intensity projection on each channel separately. For automated nuclei instance detection and segmentation in spheroids, a deep learning object detection and instance segmentation workflow incorporating Mask R-CNN [86] was implemented. The neural network was initialized using pretrained models trained on the ‘Microsoft COCO: Common Objects in Context’ dataset [87] and fine-tuned using images of nuclei acquired from various unrelated sources. Maximum intensity projections of DAPI images were used as inputs for the neural network to produce segmentation for each individual nucleus as outputs. Nuclei sizes were calculated using these segmented DAPI masks, and objects smaller than 350 pixels were filtered out and excluded from subsequent analysis.

For quantification and analysis of transcript abundance of marker mRNAs specific for stem-like (*LGR5*), Paneth-like (*DEFA5*), and Tdiff-like (*FABP1*) cells, maximum intensity projections of RNA-FISH channels were binarized using ‘Maximum Entropy’ thresholding (FIJI/ImageJ; https://imagej.nih.gov/ij/). Transcript abundance was estimated by overlaying nuclei masks on maximum projected probe channels and calculating number of pixels lying within each mask. To account for cytoplasmic fluorescence signals localized outside of nuclei masks, we expanded nuclei before quantification by morphological dilation (two iterations) as implemented in scikit-image (Python). To quantify mitochondrial abundance per cell, Mitotracker signals were quantified similarly, but binarization of fluorescence signal was based on ‘Moments’ thresholding (FIJI/ImageJ). We then performed k-means clustering on frequency distributions of pixel counts per cell to identify and separate cells into two distinct positive ‘ON’ (high expression/abundance) and negative ‘OFF’ (low expression/abundance) states. *k* = 2 was used for mRNA probes, while *k* = 3 was used for Mitotracker signals to better capture gradual differences between cells. Finally, the fraction of stem-like, Paneth-like, and Tdiff-like cells that are Mitotracker^high^ at the same time was calculated by dividing the number of Mitotracker^high^ *LGR5*^+^, *DEFA5*^+^, or *FABP1*^+^ cells by the total number of *LGR5*^+^, *DEFA5*^+^, or *FABP1*^+^ cells.

### 4.10. Flow Cytometry and Sorting of Metabolic Subpopulations

Spheroid cultures were dissociated into single-cell suspensions as described above. Sorted cells were collected in culture medium supplemented with 100 µg/mL streptomycin and 100 U/mL penicillin.

For MMP staining with Mitotracker, cells were resuspended in 25 nM Mitotracker Red CMXRos in PBS (1 mL per 10^6^ cells). Staining was performed for 30 min at 37 °C. For dead cell exclusion, cells were stained with 200 nM TOTO-3 in PBS. Cells were resuspended in PBS, filtered through a 35 µm cell strainer (Becton Dickinson, Franklin Lakes, NJ, USA), and analyzed on a cell sorter (AriaII and FACS Diva). Mitotracker signal was detected in the PE-CF594 channel (561 nm laser; 600 LP, 610/20 filter). TOTO-3 signal was detected in the APC channel (633 nm laser; 670/30 filter). Samples were gated for cells (FSC-A vs. SSC-A), singlets (FSC-A vs. FSC-H, SSC-H vs. SSC-W), and living cells (FSC-A vs. APC-A). Sorting was performed based on Mitotracker signal intensity (FSC-A vs. PE-CF594-A; Figure S6a–f).

For MMP staining with JC-1, cells were counted and resuspended in 1 µg/mL JC-1 (ThermoFisher, Waltham, MA, USA) in PBS (1 mL per 10^6^ cells). Staining was performed for 10 min at 37 °C. For dead cell exclusion, cells were stained with 200 nM TOTO-3 in PBS. Cells were resuspended in PBS, filtered through a 35 µm cell strainer, and analyzed on a cell sorter (AriaII and FACS Diva). JC-1 monomer signal was detected in the FITC channel (488 nm laser; 505 LP, 525/50 filter). JC-1 aggregate signal was detected in the PE channel (561 nm laser; 575/25 filter). TOTO-3 signal was detected in the APC channel (633 nm laser; 670/30 filter). Samples were gated for cells (FSC-A vs. SSC-A), singlets (FSC-A vs. FSC-H, SSC-H vs. SSC-W), and living cells (FSC-A vs. APC-A). Sorting was performed based on JC-1 aggregate/monomer ratio (PE-A vs. FITC-A). As negative control, 50 µM CCCP (Selleckchem, Houston, TX, USA) was added during the staining.

For ROS staining, cells were resuspended in 5 µM CellROX Deep Red Reagent (ThermoFisher, Waltham, MA, USA) in PBS (500 µL per 10^6^ cells). Staining was performed for 45 min at 37 °C. For dead cell exclusion, cells were stained with 1 µg/mL propidium iodide (Sigma-Aldrich, St. Louis, MO, USA) in PBS. Cells were resuspended in PBS, filtered through a 35 µm cell strainer, and analyzed on a cell sorter (AriaII and FACS Diva). CellROX signal was detected in the APC channel (633 nm laser; 670/30 filter). Propidium iodide signal was detected in the PE-CF594 channel (561 nm laser; 600 LP, 610/20 filter). Samples were gated for cells (FSC-A vs. SSC-A), singlets (FSC-A vs. FSC-H, SSC-H vs. SSC-W), and living cells (FSC-A vs. PE-CF594-A). Sorting was performed based on CellROX signal intensity (FSC-A vs. APC-A).

### 4.11. Assessment of SFC Frequency

For each sorted cell population (OXPHOS^low^, OXPHOS^high^), 48 wells with 10 cells, 24 wells with 100 cells, and 16 wells with 1000 cells per well were sorted into 96-well ultra-low attachment plates (Corning, Corning, NY, USA) containing 100 µL of culture medium (50% fresh, 50% conditioned (filtered medium of the bulk culture harvested during collection of cells)) supplemented with 100 µg/mL streptomycin and 100 U/mL penicillin per well. Fresh cytokines and medium were added every four days. Spheroid formation was analyzed 5–7 days after sorting using conventional light microscopy (Axiovert 40C, Zeiss, Oberkochen, Germany). Based on the fraction of spheroid-containing wells for each dilution, SFC frequencies were calculated using Poisson statistics and maximum likelihood (L-Calc, StemCell Technologies, Vancouver, BC, Canada). In vitro limiting dilution assays upon Mitotracker staining were performed three times for MMP^low^ and MMP^high^ subpopulations of P1 and P4, twice for P5 as well as bulk (all living, i.e., TOTO3^–^cells) populations of P1 and P4, and once for P2 and P10.

### 4.12. Assessment of TIC Frequency

Mitotracker stained cells were sorted as described above, pelleted, resuspended in medium, and counted. Different cell counts were pelleted, resuspended in medium, mixed with matrigel (Corning, Corning, NY, USA), and injected subcutaneously into the flanks of immunodeficient NSG mice. For MMP^low^ as well as MMP^high^ fractions of P1, four mice with 10^6^ cells, five mice with 10^5^ cells, six mice with 10^4^ cells, and six mice with 10^3^ cells were transplanted. For MMP^low^ as well as MMP^high^ fractions of P4, three mice with 3 × 10^5^ cells, four mice with 3 × 10^4^ cells, 5–6 mice with 3 × 10^3^ cells (six mice for MMP^low^, five mice for MMP^high^), 5–6 mice with 3 × 10^2^ cells (five mice for MMP^low^, six mice for MMP^high^), and six mice with 3 × 10^1^ cells were transplanted. The experiments were performed blindly until observable tumor development.

Mice were monitored daily for tumor formation and sacrificed when tumors reached the maximum tolerable size or when experiments were ended (P1: seven weeks; P4: five weeks after transplantation). Based on the fraction of tumor formation for each dilution, TIC frequencies were calculated using Poisson statistics and maximum likelihood (L-Calc).

### 4.13. Co-Cultivation Experiments

Spheroid cultures (*n* = 3) were transduced with a lentiviral vector encoding for EGFP under control of the human PGK promoter at multiplicities of infection of 0.5 (P1, P4) or 1 (P5), yielding transduction efficiencies of ~40–50%. After expansion, cells were stained with Mitotracker and prepared for flow cytometry as described above. In addition to Mitotracker and TOTO-3, EGFP fluorescence was detected (488 nm laser; 505 LP, 525/50 filter). Cells were gated for low and high Mitotracker signal (MMP^low^, MMP^high^) as well as for negative or positive EGFP signal (EGFP^–^, EGFP^+^). For each culture, a set of 5 × 10^4^ MMP^high^EGFP^+^ and 5 × 10^4^ MMP^low^EGFP^–^ cells as well as a set of 5 × 10^4^ MMP^high^EGFP^–^ and 5 × 10^4^ MMP^low^EGFP^+^ cells were sorted simultaneously. To assess sorting efficiency, sorted samples were reanalyzed by recording 1000 living cells. Sorted cells were cultivated in 24-well ultra-low attachment plates (Corning, Corning, NY, USA). Spheroid formation and EGFP signal for each sample set were observed by fluorescence microscopy (Axiovert 200, Zeiss, Oberkochen, Germany). After 21 days in culture, cells were dissociated, stained with Mitotracker, and reanalyzed by flow cytometry as described.

### 4.14. Inhibitor Treatments

To assess SFC frequencies upon pretreatment with OXPHOS inhibitors, 5 × 10^5^ tumor spheroid cells (P1, P4, P5) were seeded into two wells of 6-well ultra-low attachment plates (Corning, Corning, NY, USA). After seven days, 25 µM CCCP or DMSO (Sigma-Aldrich, St. Louis, MO, USA) were added and cells were incubated for 4 h at 37 °C. Cells were dissociated, stained with 200 nM TOTO-3 in PBS, and prepared for cell sorting as described. Living (i.e., TOTO-3^–^) cells were sorted into 96-well ultra-low attachment plates containing 100 µL of fresh culture medium supplemented with 100 µg/mL streptomycin and 100 U/mL penicillin per well. Limiting dilution and determination of SFC frequency were performed as described.

### 4.15. Immunohistochemistry

Formalin-fixed and paraffin-embedded tumor specimens of primary colorectal adenocarcinomas (*n* = 25 patients) and liver metastases (*n* = 25 patients) resected between 2013 and 2016 at the University Hospital Heidelberg were extracted from the archive of the Institute of Pathology, Heidelberg University, with the support of the tissue bank of the NCT (#2831). Tissues were used in accordance with the ethical regulations of the tissue bank of the NCT defined by the local ethics committee. Diagnoses were made according to the recommendations of the World Health Organization classification of tumors of the digestive system [88].

Immunohistochemical staining was performed as previously described [89]. In brief, tissue sections were cut, pretreated with an antigen retrieval buffer, and stained for Ki-67, CA9, and LDH-A using an automatic staining device (Ventana Benchmark Ultra, Roche, Basel, Switzerland; Table S3).

### 4.16. Mass Spectrometry

Mass spectrometry was performed for *LGR5*^+^ (i.e., *LGR5* score > 1) patient-derived spheroid cultures (*n* = 4). Tumor spheroid cells were stained with Mitotracker, prepared for sorting as described, and 5 × 10^5^ cells of MMP^low^ and MMP^high^ subfractions were sorted (*n* = 3 independent experiments). Cell pellets were reconstituted in 100 µL 0.1% RapiGest SF Surfactant (Waters, Milford, MA, USA) in 100 mM triethylammonium bicarbonate (Sigma-Aldrich, St. Louis, MO, USA) and 1× protease inhibitor cocktail (cOmplete, Sigma-Aldrich, St. Louis, MO, USA). Cells were lysed by probe-sonication twice for 15 s at 10% frequency, followed by centrifugation for 30 min at 15,000× *g* and 4 °C. 10 µg of protein per sample were denatured for 5 min at 95 °C, reduced with dithiothreitol (Biomol, Hamburg, Germany; 5 mM final concentration) for 30 min at 60 °C, and alkylated with chloroacetamide (Sigma-Aldrich, St. Louis, MO, USA; 15 mM final concentration) for 30 min at 23 °C. Proteins were digested overnight at 750 rpm and 37 °C, at an enzyme/protein ratio of 1:20 with sequencing-grade modified trypsin (Promega, Madison, WI, USA) in double-distilled water (ddH_2_O). Samples were acidified by adding trifluoroacetic acid (Biosolve Chimie, Dieuze, France; 0.5% final concentration), incubated for another 30 min at 750 rpm and 37 °C, and centrifuged for 30 min at 15,000× *g* and 23 °C.

Peptides were separated using the Easy NanoLC 1200 fitted with a trapping column (Acclaim PepMap C18, ThermoFisher, Waltham, MA, USA; 5 μm, 100 Å, 100 μm × 2 cm) and an analytical column (nanoEase MZ BEH C18, Waters, Milford, MA, USA; 1.7 μm, 130 Å, 75 μm × 25 cm). The outlet of the analytical column was coupled directly to a Q-Exactive HF Orbitrap mass spectrometer (ThermoFisher, Waltham, MA, USA). Solvent A was ddH_2_O (Biosolve Chimie, Dieuze, France), 0.1% (*v*/*v*) formic acid (Biosolve Chimie, Dieuze, France) and solvent B was 80% acetonitrile (ThermoFisher, Waltham, MA, USA) in ddH_2_O, 0.1% (*v*/*v*) formic acid. Samples were loaded and peptides eluted with a 105 min gradient via the analytical column as described [90].

Raw files were processed using MaxQuant (https://www.maxquant.org; version 1.5.1.2) [91] against the human Uniprot database (20170801_Uniprot_homo-sapiens_canonical_reviewed; 20,214 entries) using the Andromeda search engine with the default search criteria: enzyme was set to trypsin/P with up to two missed cleavages. Carbamidomethylation (C) and oxidation (M)/acetylation (protein N-term) were selected as fixed and variable modifications, respectively. Protein quantification was performed using the label-free quantification algorithm of MaxQuant. On top, intensity-based absolute quantification intensities were calculated with a log-fit enabled. Identification transfer between runs via the ‘matching between runs’ algorithm was allowed with a match time window of 0.3 min. Peptide and protein hits were filtered at a false discovery rate of 1% with a minimal peptide length of seven amino acids. The reversed sequences of the target database were used as a decoy database. Proteins only identified by a modification site, contaminants, as well as reversed sequences were removed from the dataset.

Differential expression analysis was performed using limma moderated t statistics (R package version 3.36.3; one-sample, two-sided) [92]. Here, data was first normalized based on median label-free quantification densities per sample. Next, ratios between MMP^high^ and MMP^low^ cells were calculated. Significantly differentially expressed proteins were defined to show a Benjamini–Hochberg adjusted *p*-value < 0.05 and an absolute log_2_-fold change > 1.

### 4.17. Quantification and Statistical Analysis

Statistical tests and sample size used for individual experiments are described in the corresponding figure legends or methods. The threshold for statistical significance was defined as *p* < 0.05. Significance levels were denoted by asterisks: * *p* < 0.05, ** *p* < 0.01, **** *p* < 0.0001. The threshold for statistical significance in univariable and multivariable survival analyses was defined as *p* < 0.1.

### 4.18. Data and Code Availability

scRNA-seq data have been deposited at the European Genome-phenome Archive (EGA) which is hosted at the EBI and the CRG, under accession number EGAS00001004064.

The mass spectrometry proteomics data have been deposited to the ProteomeXchange Consortium via the PRIDE [93] partner repository with the dataset identifier PXD018230.

Codes for analysis of scRNA-seq and RNA-FISH data are available at the github repository (https://github.com/eilslabs/CRC_scRNAseq).

## 5. Conclusions

In this study, we show at single-cell resolution that transcriptional heterogeneity identifies functional states during tumor-initiating cell differentiation in colorectal cancer. Targeting specific transcriptional states associated with cancer cell differentiation unravels novel potential vulnerabilities in human colorectal cancer.

## Figures and Tables

**Table 1:** Patient overview. Patient-derived colorectal cancer spheroids (P1–P12), organoids (O1–O3), xenografts (X1, X2), and primary colorectal cancer samples (T1–T3) used for single-cell RNA-sequencing. X indicates mutation, - indicates wild type. f, female; m, male; met, metastasis; MS, microsatellite; MSI, microsatellite instable; MSS, microsatellite stable; N/A, not available.

Patient	Sex	Origin	Site	Stage (UICC)	MS status	*TP53*	*APC*	*KRAS*
P1	m	liver met	Rectum	IV	MSS	X	X	X
P2	m	lung met	Caecum	IV	MSS	X	X	X
P3	f	liver met	Rectum	IV	MSS	X	X	X
P4	f	liver met	Ascending colon	IV	MSI	X	X	X
P5	f	primary	Transverse colon	IV	MSS	-	-	-
P6	f	primary	Caecum	IV	MSS	X	-	-
P7	m	liver met	Sigmoid	IV	MSS	-	X	X
P8	m	liver met	Caecum	IV	MSS	X	-	-
P9	m	primary	Rectum	IV	MSS	X	-	X
P10	m	primary	Sigmoid	IIIB	MSS	N/A	N/A	N/A
P11	m	primary	Rectum and caecum	IIIB	MSS	-	-	-
P12	m	primary	Rectum and transverse colon	II	MSI	X	X	X
O1	f	liver met	Sigmoid	IV	MSS	X	X	X
O2	f	liver met	Caecum	IV	MSS	X	X	X
O3	f	liver met	Ascending colon	IV	MSI	-	X	-
X1	m	primary	Rectum	I	N/A	N/A	N/A	N/A
X2	m	primary	Ascending colon	II	MSI	N/A	N/A	-
T1	m	primary	Sigmoid	III	N/A	N/A	N/A	N/A
T2	m	primary	Ascending colon	IV	N/A	N/A	N/A	N/A
T3	m	primary	Ascending colon	IVa	MSS	N/A	N/A	X

**Table 2:** Single-cell RNA-sequencing analysis. Top: Colorectal cancer (CRC) spheroids (P1–P12). Cultures with an *LGR5* score (=*LGR5* read counts/cell number) >1 are considered *LGR5*^+^. Bottom: patient-derived organoids (PDOs; O1–O3), patient-derived xenografts (PDXs; X1, X2), and tumors (T1–T3). Cell numbers for X1 and X2 indicate human cells. Cell numbers in brackets indicate epithelial cells used for analysis of T1–T3. *Hs*, *Homo sapiens*; *Mm*, *Mus musculus*; QC, quality control.

**CRC Spheroids**				
Patient	Mean Reads Per Cell	Cell Number after QC	Mean Detected Genes Per Cell	*LGR5* Score
P1	348,016	325	3535	12.85
P2	261,595	309	4072	0.23
P3	460,471	551	4537	6.43
P4	1,061,813	263	4186	87.72
P5	334,099	502	3943	4.61
P6	1,276,856	141	5116	0.03
P7	359,362	434	4335	10.18
P8	190,170	197	4174	3.38
P9	527,407	464	4354	0.00
P10	391,680	736	3418	3.35
P11	505,439	308	4036	1.43
P12	454,258	433	3977	0.00
**CRC PDOs, PDXs, Tumors**				
Sample	Mean Reads Per Cell	Cell Number after QC	Mean Detected Genes Per Cell (*Hs*)	Mean Detected Genes Per Cell (*Mm*)
O1	120,218	5550	5542	-
O2	169,086	3003	5425	-
O3	73,415	8785	4176	-
X1	238,836	1475	1841	2281
X2	237,891	1070	4598	2415
T1	1,333,884	362 (136)	3646	-
T2	847,472	538 (77)	4090	-
T3	623,942	724 (40)	2474	-

## Data Availability

scRNA-seq data have been deposited at the European Genome-phenome Archive (EGA) which is hosted at the EBI and the CRG, under accession number EGAS00001004064. The mass spectrometry proteomics data have been deposited to the ProteomeXchange Consortium via the PRIDE [93] partner repository with the dataset identifier PXD018230. Codes for analysis of scRNA-seq and RNA-FISH data are available at the github repository (https://github.com/eilslabs/CRC_scRNAseq). Survival analysis was based on publicly available data generated by the TCGA Research Network: https://www.cancer.gov/tcga.

## Supplementary Materials

The following are available online at https://www.mdpi.com/2072-6694/13/5/1097/s1, Figure S1: Identification of heterogeneous gene expression programs, Figure S2: Cell cycle and proliferative activity of human colorectal cancer cells, Figure S3: Single-cell expression of representative stem and differentiation markers in patient-derived tumor models, Figure S4: Spatial localization in spheroid cultures in situ, Figure S5: Metabolic heterogeneity in colorectal tumors and patient-derived spheroids, Figure S6: Gating strategy for sorting according to mitochondrial membrane potential (MMP) using Mitotracker, Table S1: Signatures defined by non-negative matrix factorization (NNMF) of colorectal cancer spheroid single-cell RNA-sequencing data, Table S2: Univariable and multivariable survival models, Table S3: Antibodies for immunohistochemistry.
