# Supplementary material for: Functional States in Tumor-Initiating Cell Differentiation in Human Colorectal Cancer
Source: Cancers (Basel). 2021 Mar 4;13(5):1097. doi: 10.3390/cancers13051097 (PMC7961698; doi:10.3390/cancers13051097)
Supplement: Supplementary file 1 [file cancers-13-01097-s001.zip › cancers-1128259supplementary.docx]

Supplementary materials

Functional States in Tumor-Initiating Cell Differentiation in Human Colorectal Cancer

Martina K. Zowada ^1,2,3,†^, Stephan M. Tirier ^4,5,6,†^, Sebastian M. Dieter ^1,2,7,†^, Teresa G. Krieger ^4,5,8^, Ava Oberlack ^1^, Robert Lorenz Chua ^4,5,8^, Mario Huerta ^1,2^_,_ Foo Wei Ten ^4,5,8^, Karin Laaber ^1,2,3^, Jeongbin Park ^4,8^, Katharina Jechow ^4,5,8^, Torsten Müller ^9,10^, Mathias Kalxdorf ^9,10^, Mark Kriegsmann ^11^, Katharina Kriegsmann ^12^, Friederike Herbst ^1,2^, Jeroen Krijgsveld ^9,10^, Martin Schneider ^13^, Roland Eils ^4,5,8,14^, Hanno Glimm ^1,2,15,16,‡,^*, Christian Conrad ^4,5,8,‡,^* and Claudia R. Ball ^1,2,15,16,‡,^*

| **Citation:** Zowada, M.K.; Tirier, S.M.; Dieter, S.M.; Krieger, T.G.; Oberlack, A.; Chua, R.L.; Huerta, M.; Ten, F.W.; Laaber, K.; Park, J.; et al. Functional States in Tumor-Initiating Cell Differentiation in Human Colorectal Cancer. *Cancers* **2021**, *13*, x. https://doi.org/10.3390/xxxxx  Academic Editor: Marta Baiocchi  Received: 12 February 2021  Accepted: 28 February 2021  Published: date  **Publisher’s Note:** MDPI stays neutral with regard to jurisdictional claims in published maps and institutional affiliations.  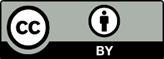  **Copyright:** © 2020 by the authors. Submitted for possible open access publication under the terms and conditions of the Creative Commons Attribution (CC BY) license (http://creativecommons.org/licenses/by/4.0/). |
| --- |

^1^ Translational Functional Cancer Genomics, National Center for Tumor Diseases (NCT) Heidelberg and German Cancer Research Center (DKFZ) Heidelberg, 69120 Heidelberg, Germany; martina.zowada@nct-heidelberg.de (M.K.Z.); sebastian.dieter@nct-heidelberg.de (S.M.D.); ava.oberlack@med.uni-muenchen.de (A.O.); mario.huerta@nct-heidelberg.de (M.H.); karin.laaber@nct-heidelberg.de (K.L.); friederike.herbst@nct-heidelberg.de (F.H.)

^2^ Department of Translational Medical Oncology, NCT Dresden and DKFZ, 01307 Dresden, Germany

^3^ Faculty of Biosciences, Heidelberg University, 69120 Heidelberg, Germany

^4^ Division of Theoretical Bioinformatics, DKFZ Heidelberg, 69120 Heidelberg, Germany; s.tirier@dkfz-heidelberg.de (S.M.T.); teresa.krieger@charite.de (T.G.K.); robert-lorenz.chua@charite.de (R.L.C.); foo-wei.ten@charite.de (F.W.T.); j.park@dkfz-heidelberg.de (J.P.); katharina.jechow@bihealth.de (K.J.); roland.eils@charite.de (R.E.)

^5^ Center for Quantitative Analysis of Molecular and Cellular Biosystems (BioQuant), Heidelberg University, 69120 Heidelberg, Germany

^6^ Division of Chromatin Networks, DKFZ Heidelberg, 69120 Heidelberg, Germany

^7^ German Cancer Consortium (DKTK), 69120 Heidelberg, Germany

^8^ Digital Health Center, Berlin Institute of Health (BIH) and Charité-Universitätsmedizin Berlin, 10117 Berlin, Germany

^9^ Division of Proteomics of Stem Cells and Cancer, DKFZ Heidelberg, 69120 Heidelberg, Germany; torsten.mueller@dkfz-heidelberg.de (T.M.); mathiaskalxdorf@gmail.com (M.K.); j.krijgsveld@dkfz-heidelberg.de (J.K.)

^10^ Medical Faculty, Heidelberg University, 69120 Heidelberg, Germany

^11^ Institute of Pathology, Heidelberg University Hospital, 69120 Heidelberg, Germany; Mark.Kriegsmann@med.uni-heidelberg.de

^12^ Department of Hematology, Oncology and Rheumatology, Heidelberg University Hospital, 69120 Heidelberg, Germany; Katharina.Kriegsmann@med.uni-heidelberg.de

^13^ Department of General, Visceral and Transplantation Surgery, Heidelberg University Hospital, 69120 Heidelberg, Germany; Martin.Schneider@med.uni-heidelberg.de

^14^ Department for Bioinformatics and Functional Genomics, Institute for Pharmacy and Molecular Biotechnology (IPMB), Heidelberg University, 69120 Heidelberg, Germany

^15^ Center for Personalized Oncology, University Hospital Carl Gustav Carus Dresden at Technische Universität (TU) Dresden, 01307 Dresden, Germany

^16^ DKTK, 01307 Dresden, Germany

* Correspondence: claudia.ball@nct-dresden.de; Tel.: +49-351-458-5536 (C.R.B.); christian.conrad@bihealth.de; Tel.: +49-30-450-543-097 (C.C.); hanno.glimm@nct-dresden.de; Tel.: +49-351-458-5540 (H.G.)

† Shared first authorship

‡ Shared last authorship

Supplementary material


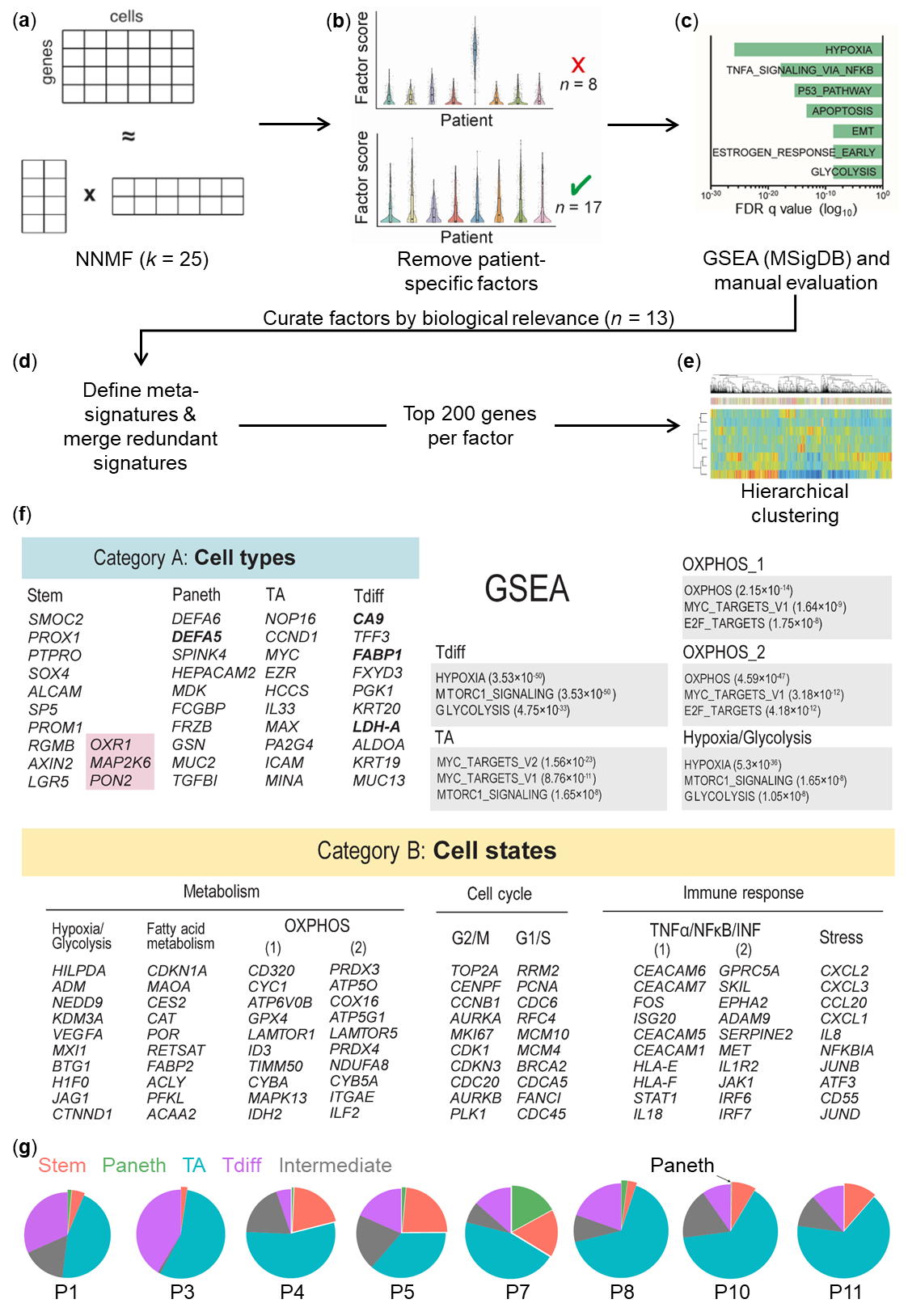


**Figure S1.** Identification of heterogeneous gene expression programs. (**a–e**) Workflow for identification of shared gene expression programs using non-negative matrix factorization (NNMF). (**a**) NNMF was performed on a normalized, mean-centered single-cell expression matrix of eight *LGR5*^+^ patients with a predicted factor number of *k* = 25. (**b**) Patient-specific factors were removed. (**c**) Top 200 genes for each factor were tested for biological relevance by gene set enrichment analysis (GSEA) and manual curation. FDR, false discovery rate; MSigDB, Molecular Signatures Database. (**d,e**) Core meta-signature scores were (**d**) defined and (**e**) clustered. (**f**) Factors (*n* = 13) identified by NNMF analysis of *LGR5*^+^ spheroid cultures (*n* = 8). Ten representative genes out of the 200 defining genes per factor are shown and ranked by NNMF factor score. Red box indicates additional stem markers playing a role in oxidative phosphorylation (OXPHOS). Markers analyzed by microscopy are highlighted in bold. Gray boxes indicate GSEA and associated FDR q values (in brackets) of hallmark gene sets for selected factors. TA, transit-amplifying; Tdiff, terminally differentiated. (**g**) Fractions of cell types (inferred by NNMF) in the *LGR5*^+^ cultures. Plots include cells (P1: *n* = 208; P3: *n* = 300; P4: *n* = 152; P5: *n* = 277; P7: *n* = 199; P8: *n* = 97; P10: *n* = 484; P11: *n* = 105) that could be assigned to one of the cell types stem-like, TA-like, Paneth-like, and Tdiff-like, or to more than one cell type (labelled as ‘intermediate’). Stem-like and Paneth-like cells are highlighted for comparability. In P3 and P11, no Paneth-like cells could be detected.


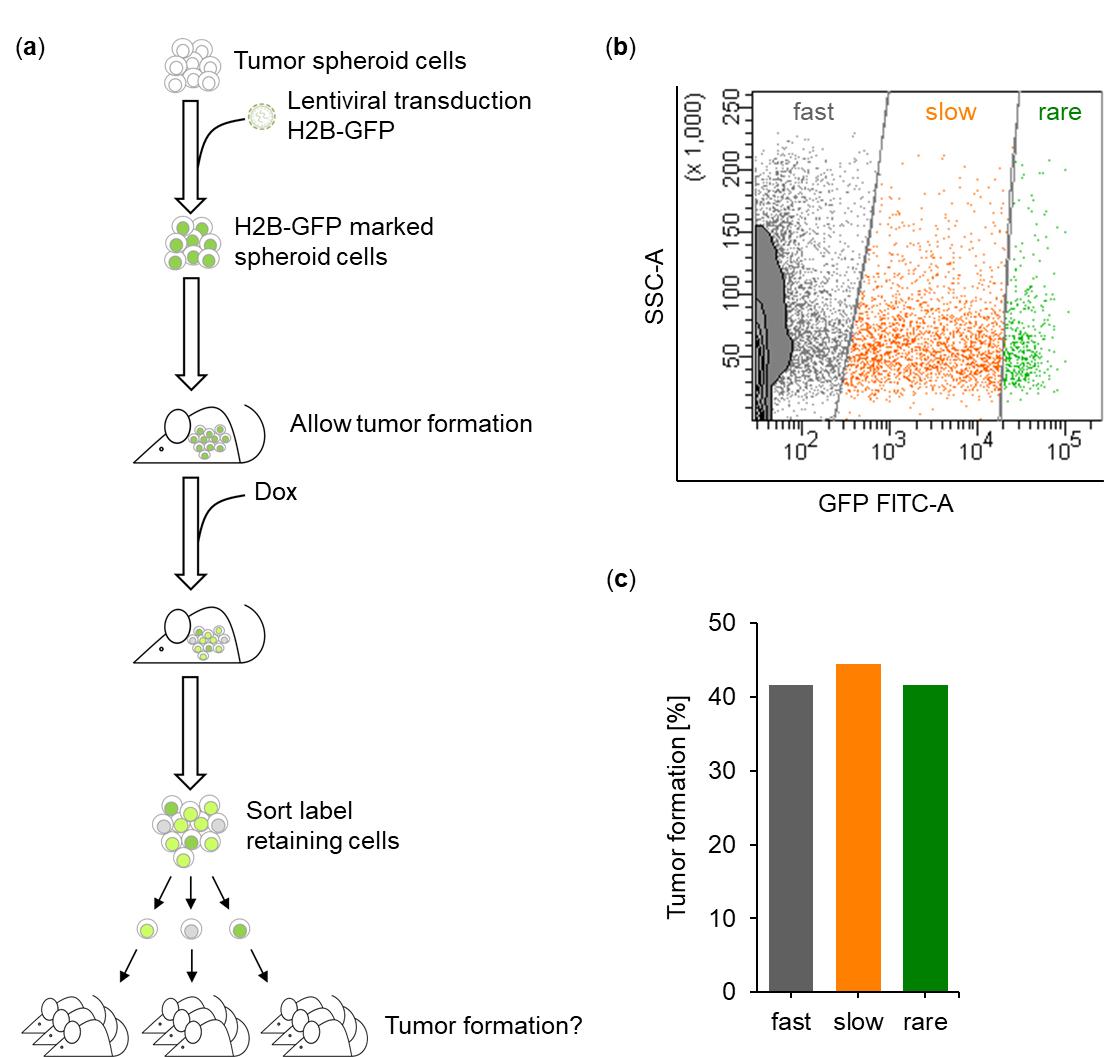


**Figure S2.** Cell cycle and proliferative activity of human colorectal cancer cells. (**a**) Experimental layout of genetic labelling experiments. Tumor spheroid cells were lentivirally transduced with histone 2 B (H2B) green fluorescent protein (GFP) and transplanted into a first generation of NOD.*Cg-Prkdc^scid^Il2rg^tm1Wjl^*/SzJ (NSG) mice. Administration of doxycycline (Dox) in mice with formed tumors led to shutdown of H2B-GFP expression. Tumors were purified and sorted according to proliferative activity as indicated by H2B-GFP expression. Sorted subpopulations were xenotransplanted into the secondary generation. (**b**) Categories of H2B-GFP expression. Representative plot (P2) is shown. (**c**) Tumor formation in secondary mice in subpopulations with different proliferative activity. Bars show the rate of tumor formation across xenografts (fast, rare: *n* = 12; slow: *n* = 9) from different patient-derived cultures (*n* = 7).


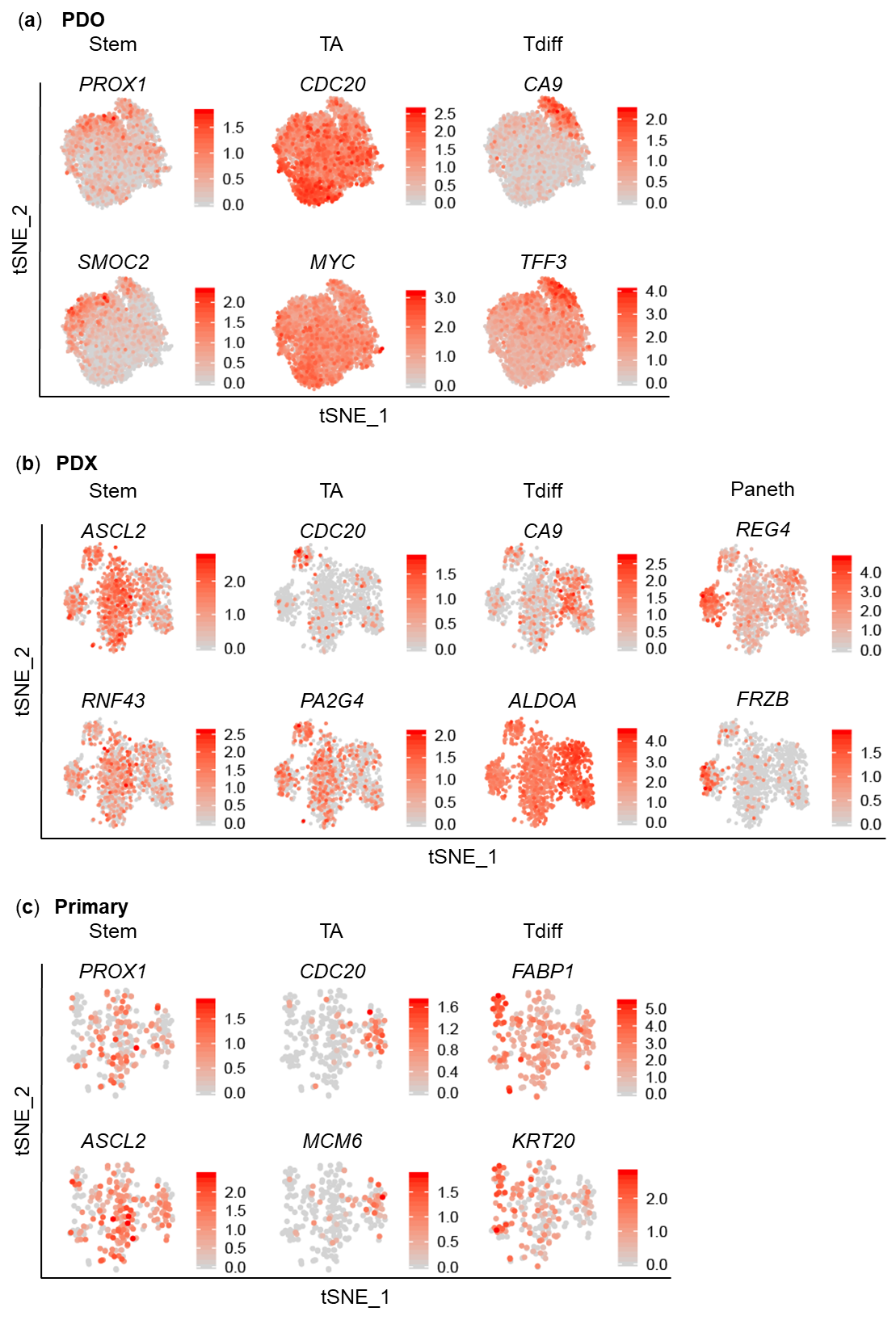


**Figure S3.** Single-cell expression of representative stem and differentiation markers in patient-derived tumor models. (**a–c**) t-distributed stochastic neighbor embedding (tSNE) depicting gene expression levels of associated markers per cell type (stem-like, transit-amplifying [TA]-like, terminally differentiated [Tdiff]-like, Paneth-like) in (**a**) patient-derived organoid (PDO; O1), (**b**) patient-derived xenograft (PDX; X1), and (**c**) merged primary tumors (*n* = 3; T1–T3).


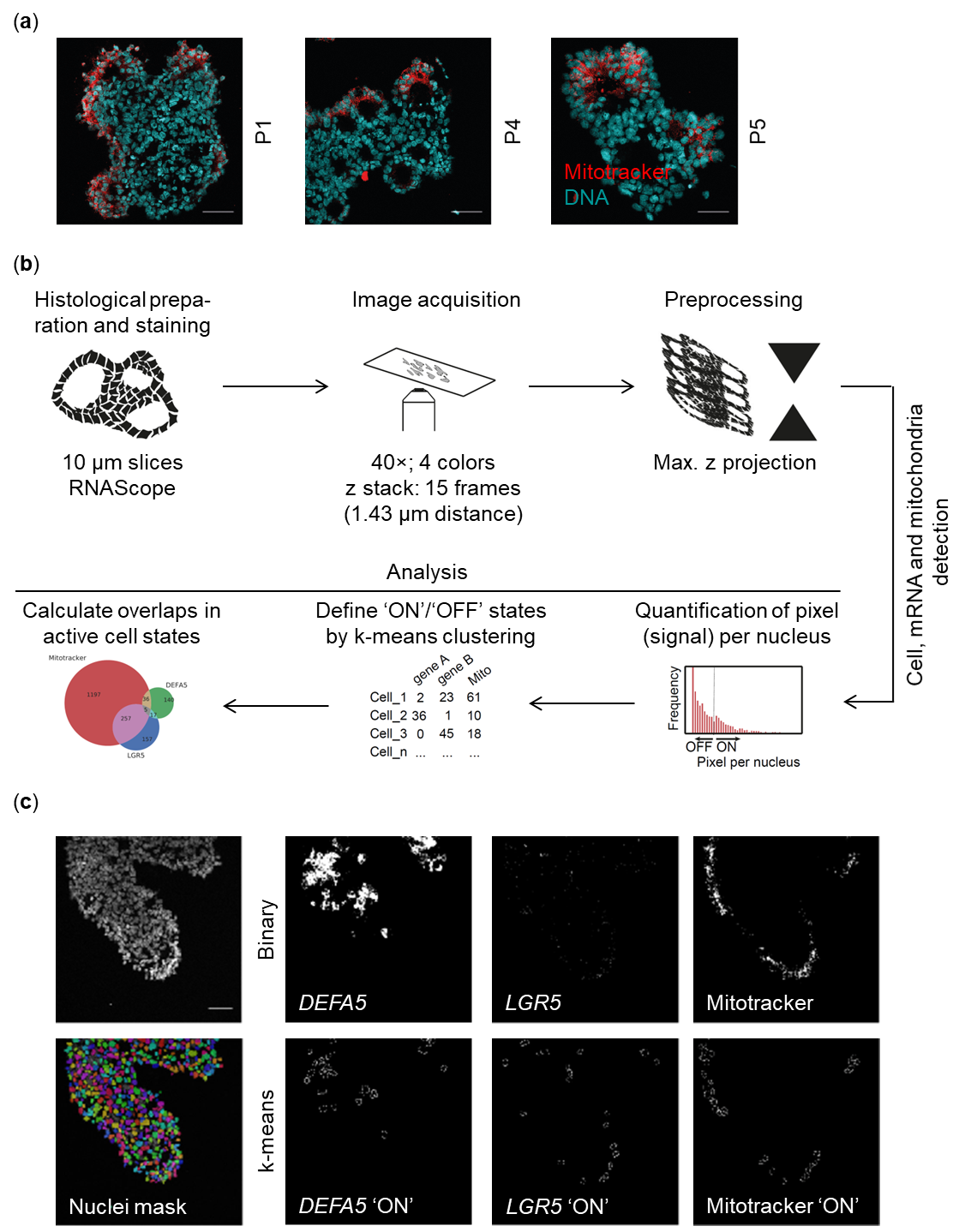


**Figure S4.** Spatial localization in spheroid cultures in situ*.* (**a**) Histological sections of tumor spheroids (*n* = 3 cultures) show maximum intensity projections of 10 µm slices stained for DNA (6’-diamidino-2-phenylindole [DAPI]) and mitochondria (Mitotracker). Scale bar, 50 µm. (**b,c**) Automated image analysis to detect active cellular states of single-cell gene expression and mitochondria. (**b**) Microscopy and image analysis workflow of combined RNA fluorescence in situ hybridization (FISH) and Mitotracker staining in spheroids. Mito, Mitotracker. (**c**) Example image for cell/nuclei detection, mRNA/Mitotracker quantification, and cell state classification. Left: DAPI signal (top) and detection of nuclei (bottom) by deep learning. Right: Binarized fluorescence signals from RNA-FISH or Mitotracker signals and corresponding ‘ON’ states (white dashed circles) as determined by k-means clustering. *DEFA5*: Paneth-like, *LGR5*: stem-like cells. Scale bar, 50 µm is representative for all images in (**c**).


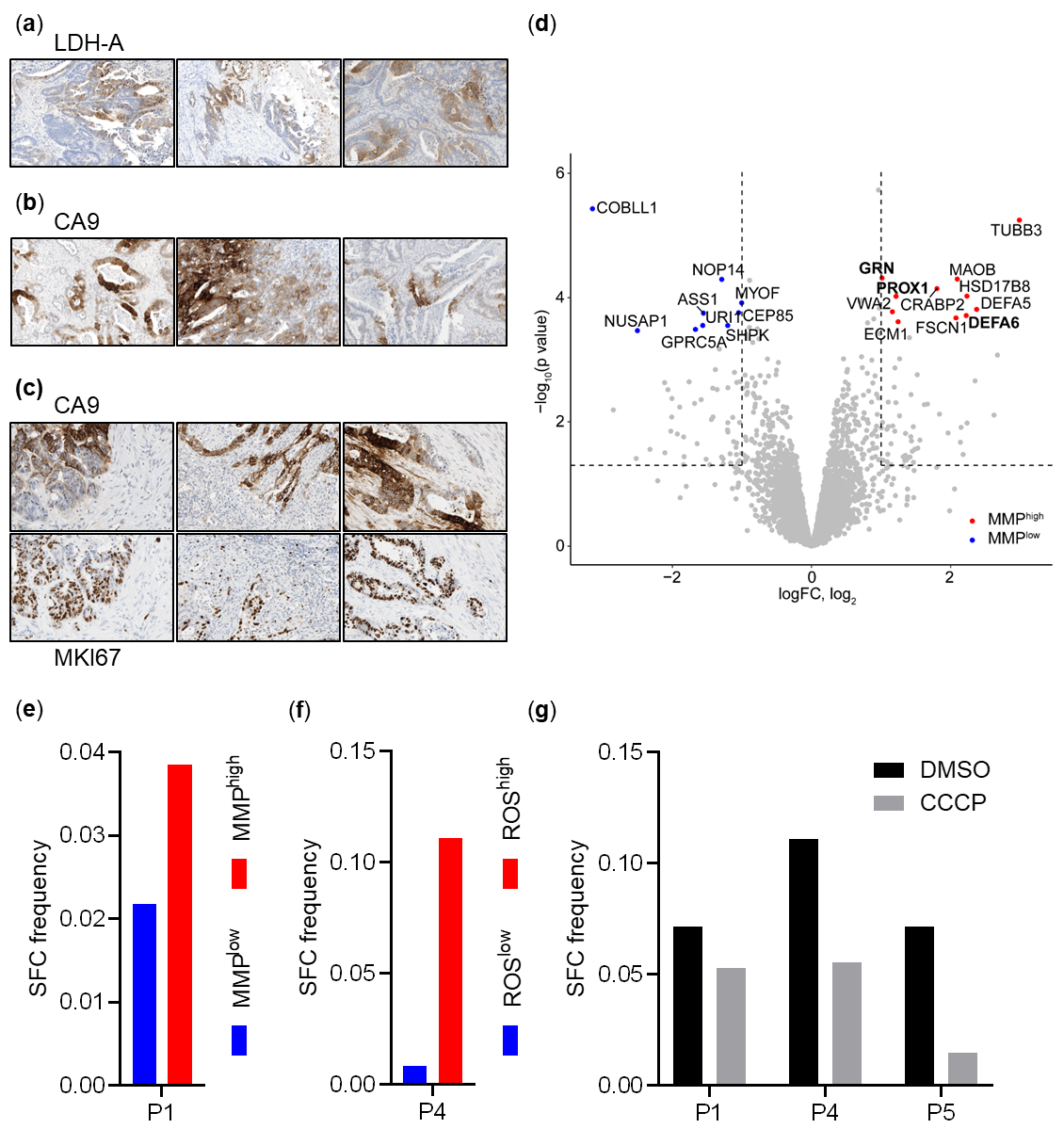


**Figure S5.** Metabolic heterogeneity in colorectal tumors and patient-derived spheroids. (**a,b**) Immunohistochemical analysis shows heterogeneous expression of (**a**) glycolysis marker LDH-A and (**b**) hypoxia marker CA9 in tumor sections from colorectal cancer patient tumors or metastases. Example pictures for three patient tumors are shown in (**a,b**). (**c**) Anti-correlative pattern of hypoxia marker CA9 and proliferation marker MKI67 expression. Example pictures for three patient tumors are shown. (**d**) Volcano plot of proteins detected by mass spectrometry of spheroid cells sorted for mitochondrial membrane potential (MMP^low^, MMP^high^). Differentially abundant proteins between sorted subpopulations of spheroid cultures (*n* = 4) after one sample t test and p value adjustment are colored. Proteins contributing from the stem signature (PROX1, GRN, DEFA6) are highlighted in bold. Cutoff borders at *p* = 0.05 and a two-fold abundance change are indicated as dashed lines. FC, fold change. (**e,f**) Spheroid-forming cell (SFC) frequencies of spheroid cells sorted for (**e**) MMP (JC-1) and (**f**) reactive oxygen species (ROS; CellROX) determined by in vitro limiting dilutions. SFC frequencies were calculated based on sphere formation seven days after seeding. (**g**) Effect of carbonyl cyanide *m*-chlorophenyl hydrazine (CCCP) pretreatment on SFC frequencies in spheroid cultures (*n* = 3). Cells were pretreated with 25 µM CCCP for 4 h and seeded for limiting dilutions. SFC frequencies were assessed seven days after seeding.


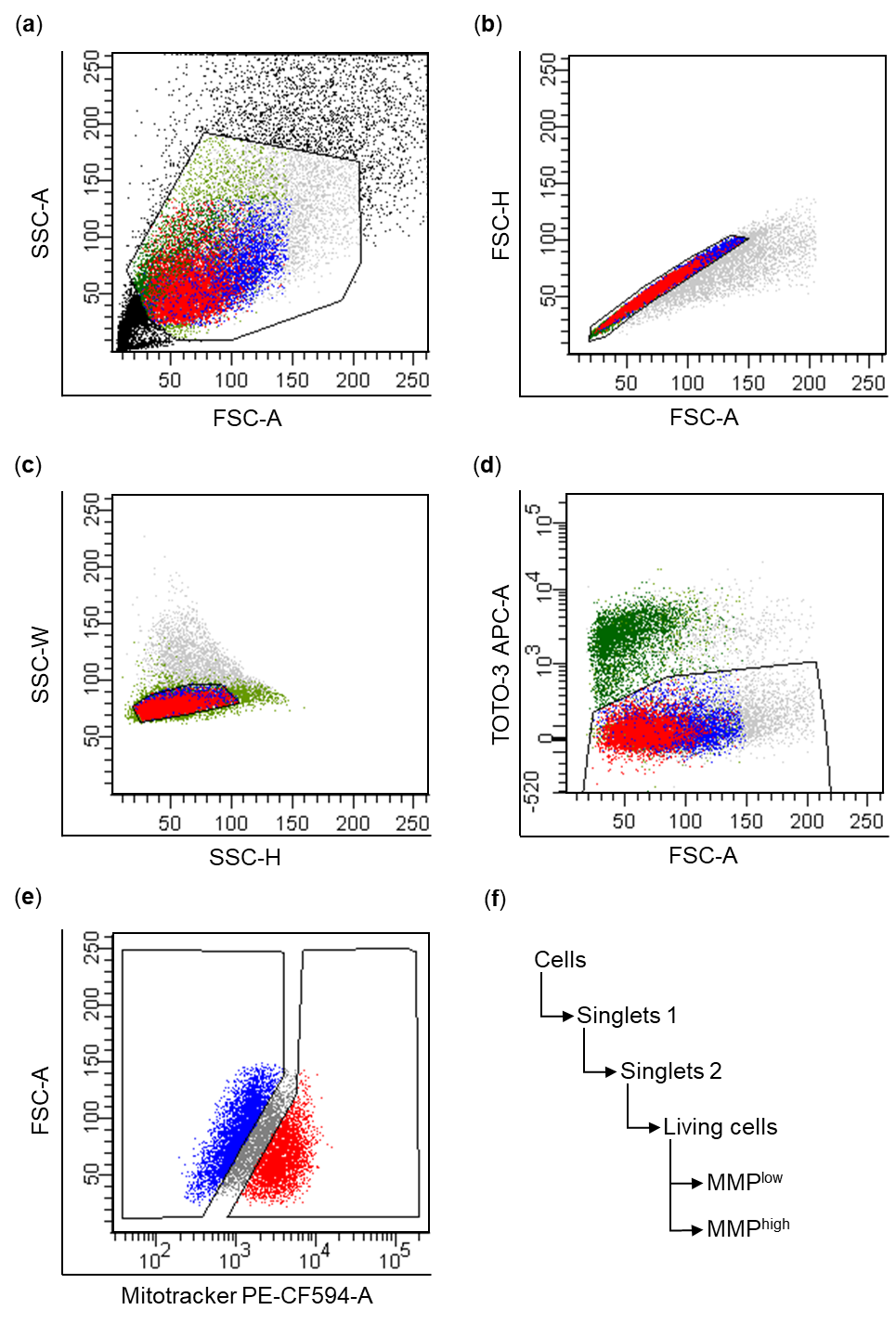


**Figure S6.** Gating strategy for sorting according to mitochondrial membrane potential (MMP) using Mitotracker. (**a–f**) Representative gating strategy (P1) is shown. (**a**) All events (black) are gated for cells (light gray). (**b,c**) Cells are gated for singlets based on (**b**) FSC (light green) and (**c**) SSC (dark green). (**d**) Singlets are gated for living cells (dark gray). y-axis is displayed biexponentially. (**e**) Living cells are gated for MMP^low^ (blue) and MMP^high^ cells (red). (**f**) Population hierarchy.

**Table S1.** Signatures defined by non-negative matrix factorization (NNMF) of colorectal cancer spheroid single-cell RNA-sequencing data. NNMF signatures (*n* = 13) are defined by the top 200 genes. Similar signatures are combined as meta-signatures (*n* = 8). Genes are ranked by NNMF factor score.

See separate file.

**Table S2.** Univariable and multivariable survival models. Survival models based on the cell type and cell state-associated clusters (cl1–cl6) and other stratifications. Shaded values indicate comparisons with *p* < 0.1. Events indicate death in overall survival and recurrence in progression-free survival. Patients with tumor site uniquely classified to left or right were included in the multivariable analysis. Underlined, new variable added to the model in the analysis of variance (ANOVA) comparison. CAF, cancer-associated fibroblast; CI, confidence interval; cl, cluster; CMS, consensus molecular subtype; HR, hazard ratio.

See separate file.

**Table S3.** Antibodies for immunohistochemistry. RTU, ready to use.

| Antibody | Company | Clone | Pretreatment | Buffer  incubation  time [min] | Antibody  incubation  time [min] | Dilution |
| --- | --- | --- | --- | --- | --- | --- |
| Ki-67 | Roche | 30-9 | Tris/Borat/  EDTA, pH 8.4 | 32 | 24 | RTU |
| CAIX | Santa Cruz | H-11 | Tris/Borat/  EDTA, pH 8.4 | 16 | 24 | 1:500 |
| LDH-A | Santa Cruz | E-9 | Tris/Borat/  EDTA, pH 8.4 | 40 | 24 | 1:250 |
